# Peroxidase gene discovery from the horseradish transcriptome

**DOI:** 10.1186/1471-2164-15-227

**Published:** 2014-03-24

**Authors:** Laura Näätsaari, Florian W Krainer, Michael Schubert, Anton Glieder, Gerhard G Thallinger

**Affiliations:** 1 Institute of Molecular Biotechnology, Graz University of Technology, Petersgasse 14, 8010 Graz, Austria; 2 Austrian Centre of Industrial Biotechnology (ACIB GmbH), Petersgasse 14, 8010 Graz, Austria; 3 Institute for Genomics and Bioinformatics, Graz University of Technology, Petersgasse 14, 8010 Graz, Austria

**Keywords:** Horseradish peroxidase, *Armoracia rusticana*, Transcriptome sequencing, *Pichia pastoris*, 454 sequencing

## Abstract

**Background:**

Horseradish peroxidases (HRPs) from *Armoracia rusticana* have long been utilized as reporters in various diagnostic assays and histochemical stainings. Regardless of their increasing importance in the field of life sciences and suggested uses in medical applications, chemical synthesis and other industrial applications, the HRP isoenzymes, their substrate specificities and enzymatic properties are poorly characterized. Due to lacking sequence information of natural isoenzymes and the low levels of HRP expression in heterologous hosts, commercially available HRP is still extracted as a mixture of isoenzymes from the roots of *A. rusticana*.

**Results:**

In this study, a normalized, size-selected *A. rusticana* transcriptome library was sequenced using 454 Titanium technology. The resulting reads were assembled into 14871 isotigs with an average length of 1133 bp. Sequence databases, ORF finding and ORF characterization were utilized to identify peroxidase genes from the 14871 isotigs generated by *de novo* assembly. The sequences were manually reviewed and verified with Sanger sequencing of PCR amplified genomic fragments, resulting in the discovery of 28 secretory peroxidases, 23 of them previously unknown. A total of 22 isoenzymes including allelic variants were successfully expressed in *Pichia pastoris* and showed peroxidase activity with at least one of the substrates tested, thus enabling their development into commercial pure isoenzymes.

**Conclusions:**

This study demonstrates that transcriptome sequencing combined with sequence motif search is a powerful concept for the discovery and quick supply of new enzymes and isoenzymes from any plant or other eukaryotic organisms. Identification and manual verification of the sequences of 28 HRP isoenzymes do not only contribute a set of peroxidases for industrial, biological and biomedical applications, but also provide valuable information on the reliability of the approach in identifying and characterizing a large group of isoenzymes.

## Background

Horseradish peroxidases (HRPs) originating from the perennial herb *Armoracia rusticana* (*Brassicaceae*) are heme-containing monomeric glycoproteins belonging to the class III plant peroxidase subfamily [1]. These versatile enzymes have traditionally been utilized as reporters in various diagnostic assays and histochemical stainings but have also gained increasing interest in other life science and biotechnological applications ranging from cancer therapeutics [2], protein engineering [3] and transgenics [1] to bioremediation [4], biosensors [5] and biocatalysis [6]. The *in vivo* functions of HRPs have not been fully elucidated owing to the estimated large number of isoenzymes (up to 42) [7], but are known to be very diverse [8] thus offering a wide range of substrate specificities and applications. Although HRP has been studied for decades and in spite of the large diversity of this enzyme family, protein engineering and heterologous expression have mainly been focused on one single isoenzyme C1A, thus neglecting the potential of all others. This was largely due to the lack of sequence information and the low efficiency of HRP expression in heterologous hosts. Commercial preparations are still extracted from the roots of *A. rusticana* and therefore consist of a mixture of various isoenzymes. The quality of these preparations varies greatly and depends on several biotic and abiotic factors, such as seasonal change or origin. Chromatographic purification is needed to isolate highly enriched isoenzyme fractions. Only very few purified isoenzymes were accessible so far and their substrate specificities and enzymatic properties are poorly characterized.

The sequences of only eight isoenzymes are currently known: six nucleotide sequences (C1A, C1B, C1C, C2, C3, N) have previously been published [9-11] and two amino acid sequences A2 (P80679) [12] and E5 (P59121) [13] have been determined. However, for decades, HRP has been known as a large group of enzymes with versatile physical properties. Already in 1966, Shannon *et al. *[14] described the physical properties of seven isoenzymes. Aibara *et al*. [15,16] characterized five neutral isoenzymes (B1, B2, B3, C1 and C2) and six basic isoenzymes (E1-E6) in 1982 and 1981, respectively. A total number of 42 peroxidase isoenzymes have been identified by isoelectric focusing in commercially available HRP preparations [7], without knowing whether these isoenzymes differ in amino acid sequence or due to different post-translational modifications.

This study has two major goals. First, we resolve the transcriptome sequence of *A. rusticana*, a perennial plant of industrial and medical importance. The availability of a large expressed sequence tag (EST) collection is crucial to support annotation in possible future *A. rusticana* genome sequencing projects. Secondly, we demonstrate the use of an efficient enzyme discovery pipeline including new generation cDNA sequencing technologies, *in silico* isoenzyme discovery and experimental sequence verification, gene synthesis and enzyme production and secretion by *Pichia pastoris* (*Komagataella pastoris*) as a straightforward approach to discover and characterize new isoenzymes from plants or other eukaryotes. Transcriptomes deliver all sequences of expressed genes, at the same time avoiding sequencing introns and providing information about all expressed exons and alternative exon junctions. Studies in *Arabidopsis thaliana*, a model species of the *Brassicaceae* family, have demonstrated the power of massively parallel transcriptome sequencing in providing high-quality representation of transcripts needed for gene discovery [17]. Similar transcriptome sequencing approaches have previously been applied, for example, in marker development, population genomics [18] and predictions of biosynthetic pathways [19-23]. Over the course of the study, the new ‘third-generation’ and ‘fourth-generation’ sequencing techniques have revolutionized the speed and cost of the transcriptome sequencing projects [24]. Hundreds of transcriptomes have been sequenced and annotated, especially by the large sequencing projects 1KP [25-27], PhytoMetaSyn [28,29] and Medicinal Plant Genomics Resource [30,31]. However, the concept of novel isoenzyme discovery from the large bulk of sequences generated by next generation sequencing (NGS) technologies needs a full pipeline of efficient tools. This is the first study using NGS transcriptome sequencing to discover, discriminate and characterize large numbers of sequence verified isoenzymes of non-model plant origin. Although a similar study was recently performed to identify fungal cellulases [32], the method described utilized combined secretome and transcriptome analyses and was only aiming to show cellulose activity of the cloned cDNA without the need of the full verified sequences to make all discovered isoenzymes available by recombinant expression. The method described in this study can be widely applied for the replenishment of the sequence data in any eukaryotic organism including fungi, plants and animal cell lines or tissues when detailed sequence and gene structure information of enzymes and isoenzymes is needed.

## Results

### Sample preparation and cDNA library generation

The high quality (RNA integrity number RIN 9.4-9.8) of the RNA samples was confirmed with an Agilent 2100 bioanalyzer. In order to include the majority of all encoded isoenzymes, a mixture of RNA from diverse plant parts, including leaves, roots, sprouts and stems, was chosen for mRNA isolation. cDNA synthesis, normalization, size selection and cloning was performed by LGC Genomics (Berlin, Germany). The normalized cDNA library was subjected to quality control experiments before using it for 454 pyrosequencing: a cDNA fragment size of over 800 bp was ensured and the normalization efficiency was verified by sequencing 96 randomly selected clones (LGC Genomics).

### Sequencing and *de novo* assembly

**Figure 1:**
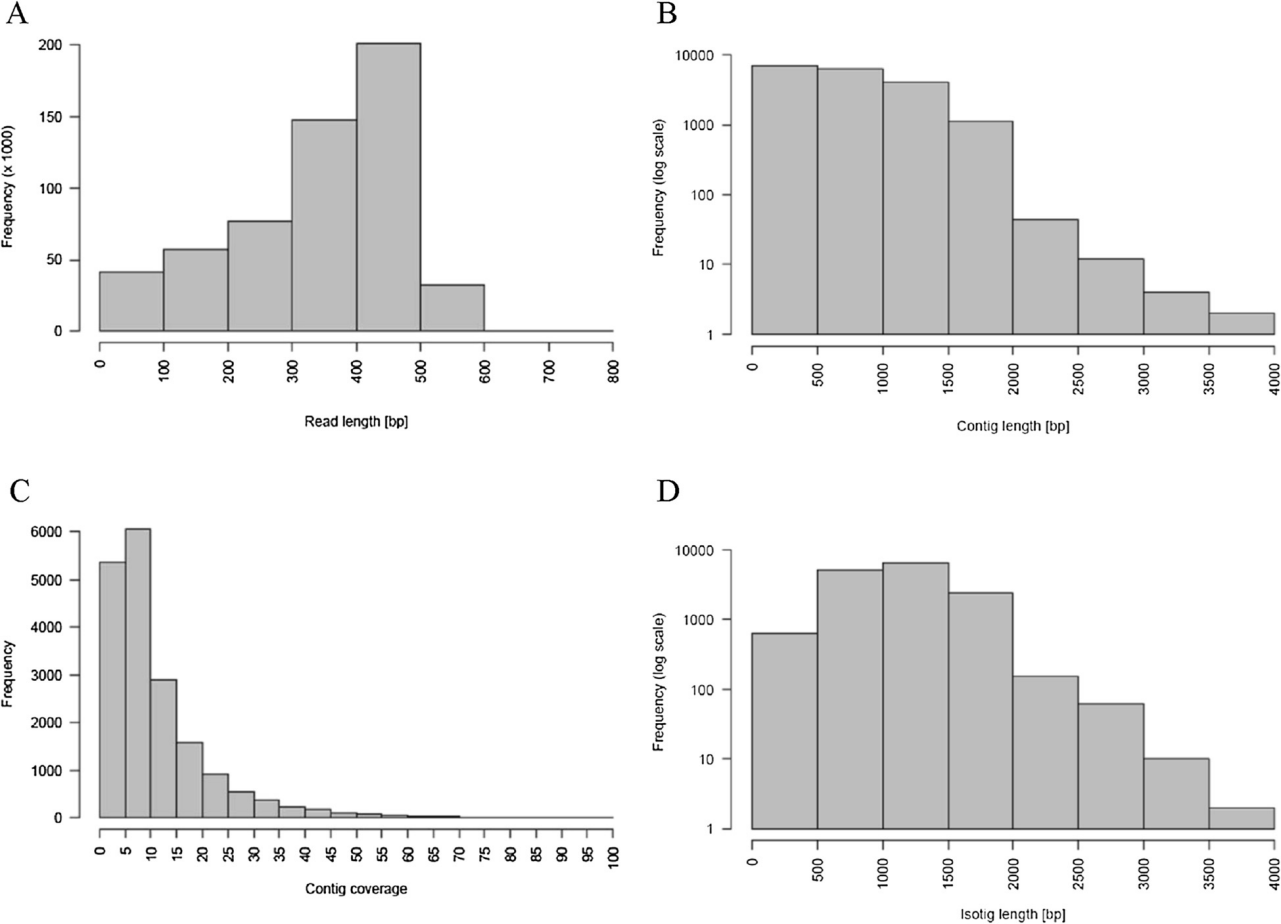
**Overview of the sequencing and assembly of the *A. rusticana *transcriptome. (A)** Size distribution of the quality-filtered reads. Total number of reads: 556269, average/median length 342.9/379.0 **(B)** Length distribution of the 18511 contigs. Average/median length of the contigs: 717.7/667.0 **(C)** Coverage distribution of the 18511 contigs. Average/median coverage of the contigs: 11.4/7.8 **(D)** Length distribution of the14871 isotigs. Average/median length of the isotigs: 1133.3/1113.0.

The normalized cDNA library was sequenced on half a picotiterplate run on the GS FLX using Roche 454/Titanium chemistry. A total of 592507 sequence reads with an average read length of 353 ± 122 nucleotides were obtained. A total of 12798 (2.16%) clonal reads (exact, 3’ or 5’) were detected. Prior to assembly, the sequence reads were screened for the linker sequence used for concatenation, the linker sequences were clipped and the reads were quality checked (LGC Genomics). The resulting 556269 reads with an average length of 343 bp (Figure 1A) were further filtered by Newbler sequence filtering to ensure consistent high quality of the reads used in the assembly. 490285 reads were aligned to individual transcripts using Newbler version 2.5.3 with default settings. The *de novo* assembly generated 18511 contigs with an average length of 718 bp (Figure 1B) and an average coverage of 11.4-fold (Figure 1C). The contigs were further processed to 14871 isotigs with an average length of 1133 bp (Figure 1D). 35950 reads were left as singletons. A detailed summary of the alignment and assembly process is described in Table 1.

**Table 1:** Summary of the transcriptome sequencing, assembly and enzyme discovery results

	**Number of sequences**	**% of total**
**Transcriptome sequencing and assembly**		
Total number of reads	592,507	-
Clipped reads	556,269	-
Reads after Newbler quality control	543,439	100.0
Reads aligned	490,285	90.2
Reads assembled	433,179	88.4
Reads partially assembled	57,064	10.5
Singletons	35,950	7.3
Contigs	18,511	-
Isogroups	10,619	-
Isotigs	14,871	-
Average isotig length	1,133	-
Largest isotig length	3,659	-
Average contig coverage	11.4	-
**HRP isoenzyme discovery and verification**		
# of isotigs with a secretory peroxidase domain	18	-
# of full length peroxidase genes	18	-
# of peroxidase genes after manual revision	20	-
Total # of isoenzymes (including allelic variants)	28	100
Successfully verified from gDNA	26	89.7
**Enzyme production in *Pichia pastoris***		
Synthetic genes for production	26	89.7
Successful production of an active isoenzyme	22	75.9

### Plant peroxidase search and manual validation of assembled transcripts

The position-specific scoring matrix (PSSM) corresponding to known horseradish peroxidases (cd00693) was used in a tblastn search in the assembled transcripts. The settings allowed for a very permissive filtering of putative peroxidases, thus including many false positives, but avoiding the loss of valuable data for further analyses. This search yielded hits in 91 transcripts, which were classified in secretory peroxidases, ascorbate peroxidases, glutathione peroxidases and peroxidase-like proteins by definition of the Conserved Domains Database (CDD, NCBI [33]). All previously known HRPs were classified as secretory peroxidases, so only the contigs comprising a secretory peroxidase conserved domain were kept. The horseradish transcriptome contigs of the 18 resulting secretory peroxidases were manually reviewed. In this process, the coding sequences of four contigs were extended with available assembly data, three contigs were split because of strongly conflicting reads, and two more contigs were discarded because of only a partial domain match that could not be resolved into a full-length sequence. In total, 20 *HRP* genes, with allelic variants corresponding to 28 peroxidase isoenzymes, were identified in the transcriptome of *A. rusticana.* This includes isoenzymes C1A and C3 which could be partially retrieved from the raw reads although they did not form a full-length contig. No read - even partially - corresponding to the previously published “neutral isoenzyme” N (Q42517) [11] was found.

### Sanger sequencing and genome walking

Sequences yielded by the transcriptome assemblies are not necessarily error free but can include incorrect information either caused by the transcription and RNA editing machineries of the plant [34,35], introduced in the sequencing process or resulting from misassemblies. The sequences of all peroxidase genes detected in the isotigs were verified on genome level by Sanger sequencing of amplified genomic DNA. In addition, the sequences of the isoenzymes C1A and C3 available in the databases and partially also found in the raw reads were revised. In case of five full-length contigs where no sufficient read data from untranslated regions was available to enable amplification and sequencing of the complete gene, a genome walking approach was successfully performed in order to verify the 5’ and/or 3’ regions of the respective gene. Divergent coding sequence information was observed for ten genes in the form of possible allelic variants. PCR artifacts were ruled out by repeated experiments to increase the coverage of the positions. Thus, conflicting sequence information was postulated not to be due to sequencing errors, but rather due to the high sequence similarity of the HRP isoenzymes and the permissive settings used in the assembly. Supporting this assumption, putative allelic sequences could be found as separate raw reads. For altogether nine positions in contigs 22684, 6117, 17517 and 23190 (Table 2) the nucleotide present in the transcriptome reads could not be found in the genomic DNA sequenced. The sequence of the previously published “neutral isoenzyme” N (Q42517) not present in the transcriptome sequences could not be amplified from genomic DNA (gDNA) either. Therefore, it was not included in the following analyses or experiments. The sequences of the transcript # 22489 (see Table 2) could not be verified.

**Table 2:** Comparison of the HRP isoenzyme sequences between GenBank, UniProt, transcriptome and verified genome sequences

**HRP**	**Sanger sequence**		**Transcriptome sequence**		**GenBank sequence**		**UniProt sequence**
	**Nt**	**aa (exon)/intron**	**nt**	**aa**	**nt**	**aa (exon)/intron**	**aa**
C1A	TA	Y37	-	-	AT	I37	Y37
	109-110				109-110		
	C1159	intron	-	intron	G1159	intron	-
C1B	T/C253	intron	-	intron	T253	intron	-
	T/C859	intron	-	intron	C859	intron	-
C1C	ss	ss	ss	ss	*	*	*
	nt1-60	aa1-20	nt1-60	aa1-20			
	C178	R60	C178	R60	A118	S40	S40
	A/T1335	intron	-	intron	-	-	-
	A/G1888	T/A165	A/G493	T/A165	G433	A145	A145
	C1889	A165	C/T1889	A/V165			
	C/G1921	Q/E176	C/G526	Q/E176	G466	E156	E156
C2	CT	intron	-	intron	*	intron	-
	1250-1251						
	A1334	intron	-	intron	*	intron	-
C3	G/T1294	intron	-	intron	G1294	intron	-
	A/T1323	intron	-	intron	A1323	intron	-
	T/C1484	L231	-	-	T1484	L231	L231
	C/T1541	F250	-	-	C1541	F250	F250
A2	ss	ss	ss	ss	-	-	*
	nt1-93	aa1-31	1-93	aa1-31			
	AAT	N78	AAT	N47	-	-	D47
	231-234		231-234				
	GGA	G220	GGA	G220	-	-	N189
	996-998		661-663				
	AAT	N221	AAT	N221	-	-	G190
	999-1001		664-666				
	ACG	T284	ACG	T284	-	-	L253
	1185-1187		850-852				
	G/A1203	A/T290	G868	A290	-	-	A259
	AAT	N334	AAT	N334	-	-	D303
	1335-1337		999-1002				
E5	ss	ss	ss	ss	-	-	*
	nt1-81	aa1-27	nt1-81	aa1-27			
	T419	L82	C/T246	L82	-	-	L55
	C422	D83	T/C249	D83	-	-	D56
	C545	C124	T/C372	C124	-	-	C97
01805	None	none	none	none	-	-	-
22684	G1611	R337	A1010	K337	-	-	-
	TGA	D343	CGG	G343	-	-	-
	1627-1629		1026-1028				
01350	None	none	none	none	-	-	-
02021	None	none	none	none	-	-	-
03523	None	none	none	none	-	-	-
06117	T30	V10	C/T30	V10	-	-	-
	C1088	I269	T807	I269	-	-	-
17517	T190	Y64	C190	H64	-	-	-
	C1157	G282	T846	G282	-	-	-
	A1232	K307	G921	K307	-	-	-
08562.1	None	none	none	none	-	-	-
08562.4	None	none	none	none	-	-	-
23190	T1345	S109	G1345	S109	-	-	-
	C1423	G135	T1423	G135	-	-	-
	T1842	S222	T/C1842	S/P222			
	C1850	T224	A/C1850	T224	-	-	-
	A2221	E348	T/A2221	V/E348	-	-	-
04663	None	none	none	none	-	-	-
06351	None	none	none	none	-	-	-
05508	G/A346	A/T116	G/A346	A/T116	-	-	-
22489	-	-	G/A597	T199	-	-	-
	.	.	G/T715	A/S239	-	-	-

All nucleotide (nt) and amino acid (aa) positions were calculated from the start ATG. Variations between the nt positions of the transcriptome sequence compared to other sequence sources are either due to deletions or intronic sequences in the other sources. “-“ indicates that no sequence information is available from the respective source. “ss” indicates a putative signal sequence. Deletions or missing sequences are marked with “*”. “N/NX” indicates a variation at position X. “None” indicates that no polymorphisms or differences between transcriptome and genome sequence were found. The isoenzymes C1A and C3 were detected in the transcriptome raw reads with only partial/low coverage (0-2x), thus no consensus sequence was formed.

### GC content and codon usage

**Figure 2:**
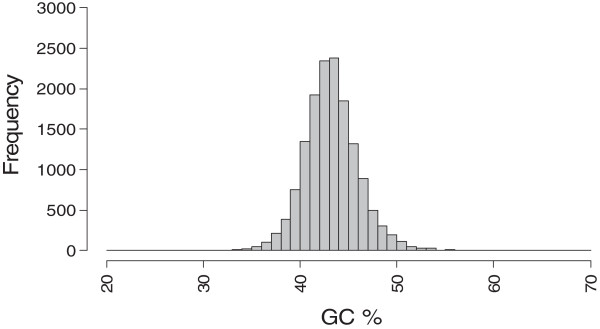
**GC content distribution of the *A. rusticana *isotigs.** GC content distribution of the *A. rusticana* isotigs varies from 28% (min) to 62% (max) with a range of 35%. The average GC content of all isotigs is 42.72%. Mode (x-axis value) 43, mode value (y-axis value) 2376.

The average GC content of all 14871 isotigs was calculated to be 42.7% (range 28%-62%) (Figure 2), almost identical to the average GC content, 42.5%, reported for *A. thaliana *[36]. This is lower than the average GC content of 45.1% reported by the Codon Usage Database (CUD) [37], suggesting that the small set of available *A. rusticana* genes (14 coding sequences) used in CUD for the calculation is not representative for the whole species. The GC content of the HRP isoenzymes was observed to vary between 42.9% (C2, contig #04627) and 51.0% (contig #22489), with an average GC content of 47.1%. The results of the analysis are described in more detail in Table 3.

**Table 3:** Summary of the horseradish peroxidase isoenzymes and associated data produced during this study

**Contig number**	Name (* novel)	**Length nt**	**GC content%**	**Accession #**	**CAI calculated**	**Intron #**	**Signal sequence length**	**Disulfide bridges (EDBCP prediction)**	**pI mature protein**
-	C1A	1062	43.69	HE963800	0.812	3	30	[11–91] [44-49] [97–301] [177–209]	5.59
15901	C1B	1056	43.94	HE963801	0.808	3	28	[11–91] [44-49] [97–301] [177–209]	5.84
25148	C1C*	1059	45.14	HE963802	0.809	3	29	[11–91] [44-49] [97–301] [177–209]	6.49
25148_2	C1D*	1059	45.04	HE963803	0.810	3	29	[11–91] [44-49] [97–301] [177–209]	7.04
04627	C2	1044	42.91	HE963804	0.800	3	24	[11–91] [44-49] [97–301] [177–209]	8.56
-	C3	1050	46.76	HE963805	0.781	3	29	[11–91] [44-49] [97–300] [177–209]	7.71
Manual assembly	A2A*	1011	46.79	HE963806	0.761	3	31	[11–91] [44-49] [97–299] [176–208]	4.93
Manual assembly	A2B*	1011	46.69	HE963807	0.761	3	31	[11–91] [44-49] [97–299] [176–208]	4.93
04382	E5	1044	46.07	HE963808	0.771	3	27	[11–91] [44-49] [97–300] [177–209]	8.84
01805	1805*	1065	44.41	HE963809	0.797	3	31	[11–91] [44-49] [97–301] [177–209]	5.97
22684	22684.1*	1050	46.76	HE963810	0.770	3	29	[11–91] [44-49] [97–300] [177–209]	6.98
22684_2	22684.2*	1050	46.67	HE963811	0.772	3	29	[11–91] [44-49] [97–300] [177–209]	6.37
01350	1350*	975	50.97	HE963812	0.707	3	28	[11–91] [44-49] [97–292] [176–201]	8.67
02021	2021*	996	46.08	HE963813	0.788	3	29	[11–89] [44-49] [95–297]	9.46
23190	23190.1*	1080	49.26	HE963817	0.724	2	31 42	[11–92] [44-49] [98–293] [178–205] or [22–103] [55-60] [109–304] [189–216]	8.40 6.58
23190_2	23190.2*	1080	49.17	HE963817	0.722	2	31 42	[11–92] [44-49] [98–293] [178–205] or [22–103] [55-60] [109–304] [189–216]	8.60 7.09
04663	4663*	1077	47.82	HE963814	0.748	3	31	[11–91] [44-49] [97–299] [176–208]	4.48
06351	6351*	945	43.39	HE963816	0.786	3	18	[17–96] [50-55] [102–292] [180–206]	6.37
03523	3523*	960	44.58	HE963820	0.761	0	22	[11–92] [44-49] [98–293] [177–203]	8.99
05508	5508.1*	966	49.28	HE963815	0.735	2	24 30	[11-87] [44-49] [93–287] [171–198] or [17–93] [50-55] [99–293] [177–204]	8.49 8.47
05508_2	5508.2*	966	49.38	HE963815	0.735	2	24 30	[11-87] [44-49] [93–287] [171–198] or [17–93] [50-55] [99–293] [177–204]	8.49 8.47
22489_1	22489.1*	978	51.02	HE963818	0.726	2	23 34	[22–98] [55-60] [104–298] [182–209] or [11-87] [44-49] [93–287] [171–198]	8.93 8.51
22489_2	22489.2*	978	50.82	HE963819	0.727	2	23 34	[22–98] [55-60] [104–298] [182–209] or [11-87] [44-49] [93–287] [171–198]	8.93 8.51
06117	6117*	1008	47.02	HE963821	0.802	3	22 32	[11–91] [44-49] [97–298] [176–208] or [21–101] [54-59] [107–308] [186–218]	5.52 6.16
17517_1	17517.1*	972	48.46	HE963822	0.737	2	23 24	[12–88] [45-50] [94–296] [171–203] or [11-87] [44-49] [93–295] [170–202]	9.49 9.39
17517_2	17517.2*	972	48.56	HE963823	0.739	2	23 24	[12–88] [45-50] [94–296] [171–203] or [11-87] [44-49] [93–295] [170–202]	9.52 9.41
08562_1	08562.1*	996	47.09	HE963824	0.779	3	22 28	[11–91] [44-49] [97–298] [176–208] or [17–97] [50-55] [103–304] [182–214]	9.01 9.03
08562_4	08562.2*	996	47.79	HE963825	0.788	3	22 28	[11–91] [44-49] [97–298] [176–208] or [17–97] [50-55] [103–304] [182–214]	9.00 9.02

The nucleotide sequences of 28 isoenzymes were submitted to EMBL. “*” indicates novel. “CAI” = codon adaptation index. If signal sequence predictions gave more than one alternative result, both signal sequence lengths are shown, separated by “/”. Disulfide bridges were predicted using both alternatives of the mature protein (EDBCP = Ensemble-based Disulfide Bonding Connectivity Pattern prediction server).

The codon usages of the *HRP* genes and the previously known *A. thaliana* peroxidases were compared in the form of heatmaps (Additional file 1), depicting the fold change of the codon usage frequencies compared to the expected (1/64) frequency (ΔRSCU). The clustering of the isoenzymes according to their codon usage frequencies situated newly discovered isoenzymes with most divergent sequence and gene structure (*HRP*_3523, *HRP*_5508, *HRP*_22489, *HRP*_17517, *HRP*_23190) also furthest away from the previously known group C isoenzymes.

### Gene structure and phylogenetic analyses

**Figure 3:**
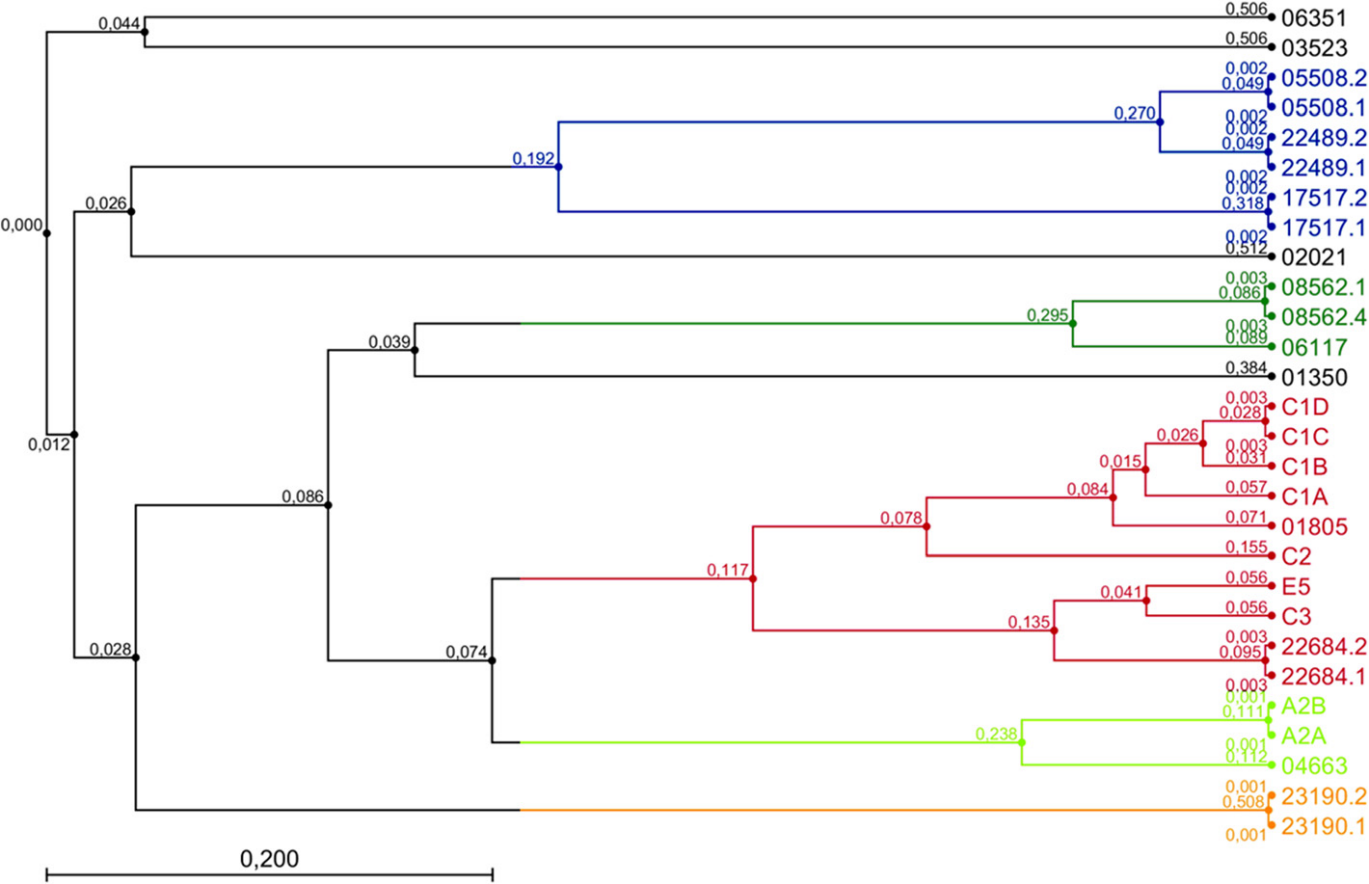
**Cladogram of all isoenzymes known and discovered during this study.** The dendrogram was cut at a branch length of 0.21 and the resulting sub-trees were colored. All previously described isoenzymes are located in the red and light green trees, respectively; whereas all novel isoenzymes (except for 01805, 22684.1, 22684.2, and 04663) cluster in distinct sub-trees (black, blue, dark green and orange) indicating a larger sequence diversity.

Phylogenetic relationships of the HRP isoenzymes are shown in Figure 3 and Additional file 1. Interestingly, the previously known isoenzymes seem to be closely related to each other, while most of the new isoenzymes discovered in the transcriptome seem to share higher evolutionary distance to them. From the 20 peroxidase gene loci, 15 were confirmed to have three introns by comparing either transcript data or protein sequence data to the verified gDNA sequence. Further four genes (5508, 22489, 17517, 23190) were noted to have only two introns and one gene (3523) no introns (Table 3). The number of introns correlates with the evolutionary distance so that genes having aberrant intron numbers were situated in separate branches close to each other in the phylogenetic topology. With the information obtained from the reads, no alternative splicing could be shown. According to SignalP, all of the isoenzymes have an N-terminal signal sequence varying in length from 18 amino acids to 31 amino acids. The lengths of the signal sequences are described in Table 3, and an alignment of the amino acid sequences of the HRP isoenzymes is shown in Additional file 2.

### Heterologous protein production in *Pichia pastoris*

Twenty-six *HRP* sequences including allelic variants were codon optimized for *P. pastoris* expression and ordered as synthetic fragments. Twenty-two of them showed activity with at least one of the substrates tested, thus verifying a successful expression in *P. pastoris*. The peroxidase activities of the produced isoenzymes were detected with four substrates having variable assay pH optima. The substrate or pH-specific performance (Table 4) of the isoenzymes suggests a wide range of possible applications for this versatile group of peroxidases.

**Table 4:** Summary of isoenzyme expression and characterization

**Contig number**	Name (* previously unknown)	**Activity ABTS**	**Activity TMB**	**Activity guaiacol**	**Activity pyrogallol**
-	C1A	+	+	+	+
15901	C1B	-	+	-	-
25148	C1C*	+	+	(+)	+
25148_2	C1D*	+	+	(+)	+
04627	C2	+	+	+	+
-	C3	+	+	+	+
Manual assembly	A2A*	+	+	+	+
Manual assembly	A2B*	+	+	(+)	(+)
04382	E5	+	+	+	+
01805	01805* (B1?)	+	+	-	-
22684	22684.1* (B2A?)	+	+	-	-
22684_2	22684.2* (B2B?)	+	+	-	-
01350	01350*	+	+	-	-
02021	02021*	-	-	-	-
23190	23190.1*	-	-	-	-
23190_2	23190.2*	n.d.	n.d.	n.d.	n.d.
04663	04663.1*	+	(+)	-	-
06351	06351*	+	+	+	+
03523	03523*	-	-	-	-
05508	05508.1*	+	+	+	+
05508_2	05508.2*	n.d.	n.d.	n.d.	n.d.
22489_1	22489.1*	+	+	(+)	+
22489_2	22489.2*	+	+	(+)	+
06117	06117*	-	-	-	-
17517_1	17517.1*	+	-	-	-
17517_2	17517.2*	+	+	+	+
08562_1	08562.1*	+	+	-	-
08562_4	08562.2*	+	+	-	+

Isoenzymes showing obvious peroxidase activity with the assay used are marked with “+”. Isoenzymes showing very low but detectable peroxidase activity with the assay used are marked with “(+)”. Isoenzymes with no activity detected during an observation period of 2 h are marked with “-“. Allelic variants not produced heterologously are marked with n.d. (no data available). Isoenzymes discovered during this study are marked with “*”.

## Discussion

Although high throughput sequencing technologies and bioinformatics tools to handle the enormous amount of data generated have been rapidly developing in the recent past, the expressed sequence data of many organisms of wide importance are still not available. Our study demonstrates how NGS technologies can provide a rapid, low-cost basis for the discovery of isoenzymes required for specific industrial, medical or biological applications. Below we discuss the reliability of the approach in identifying and characterizing an important group of isoenzymes, challenges provided by the library generation, sequencing and assembly methods, and suitability of the data obtained from the pipeline for heterologous protein production without laborious manual verification of the sequences.

A normalized cDNA collection originating from multiple *A. rusticana* tissues was sequenced with 454 pyrosequencing technology. In comparison to the other NGS methods, 454 pyrosequencing produces long reads. This is of advantage when sequencing genes coding for isoenzymes and identifying exonic variants. Longer reads increase the probability to uniquely align a given read, which might be problematic with the short reads produced by other NGS methods [38]. Studies in *A. thaliana* suggested that with the high coverage attainable by massively parallel sequencing, all transcripts can be well represented in the sequence data regardless of expression levels [17]. However, the benefits of normalization in non-model species have not been well characterized, and a recent study by Cirulli *et al*. reports low identification rates of exonic SNVs (single nucleotide variations) in non-normalized transcriptome, if the genes of interest are not well-expressed in the source tissue [38]. Since also the genome and transcriptome sizes of *A. rusticana* and the sequence data required to reach also isoenzymes with low expression levels are unknown, the cDNA library sequenced in this study was normalized. Normalization of the cDNA has been reported to be especially important in gene discovery when the cDNA used for sequencing is pooled from many tissues or individuals [39-41], and to considerably reduce the frequency of abundant transcripts thus increasing the possibility to reach also unique transcripts of isoenzymes with low expression levels. Half a plate run on a GS FLX platform resulted in over 500000 high-quality reads, corresponding to a relatively high average coverage (>10×) of the assembled 14871 isotigs with a high average length of 1133 bp. Comparable to previous transcriptome sequencing studies [40,41], 88% of the reads could be assembled into contigs. Many of the remaining singletons were of high quality and also represented an important source of information [18]. Singletons could either result from 454 sequencing errors or contaminants from plant parasites [42], they could be caused by over-efficient normalization methods, or simply represent rare transcripts with thin coverage despite the normalized cDNA pool used for sequencing.

The 454 pyrosequencing technique generates relatively long reads including very few technical errors (mainly related to homopolymer runs), and is therefore well-suited for applications such as *de novo* transcriptome sequencing. Although the sequence length achieved by 454 Titanium FLX platform is still clearly shorter than by traditional Sanger sequencing, it has been reported to be adequate for reconstructing full length transcripts [17] and validated to be comparable in accuracy to Sanger sequencing [43,44]. The high average isotig length of 1133 bp and the assembly of 90% (18/20) full length peroxidase genes reached in this study supports the statement of adequate read length and coverage to detect complete transcripts. Only isoenzymes C1A and C3 were not assembled into a contig due to low sequence coverage.

Although the error rates associated with NGS methods have been reported to be low, they could still cause problems in reliable sequence polymorphism detection. The requirement of >90% match used in this study, combined with a minimal match length of 40 bp was expected to provide a very high number of contigs without collapsing and joining similar isoenzymes into a single contig [18]. However, the isoenzyme sequences were known to be partially almost identical. To validate the assembly and ORF (open reading frame) prediction correctness, and the existence of allelic variants, the isoenzyme sequences were amplified from genomic DNA. The combination of transcriptome sequencing and Sanger sequencing of amplified genomic DNA revealed 38 variable positions in the coding sequences of 11 genes. For nine positions thereof (in four transcripts) the corresponding nucleotide could not be found in the genomic DNA. Manual confirmation of the transcriptome reads revealed that the coverage of all positions was high and that the reads agreed. Closer analysis of the positions also ruled out false variations caused by too high coverage. At relatively low or moderate levels of coverage, sequencing mistakes are not pushed over. Too high sequencing depth increases noise and could have introduced, without very strict quality control, false variations [38]. Therefore, sequencing errors could be excluded as the source of the differences. The differences could be caused either by mismatches introduced by the reverse transcriptase enzyme during library generation, as a result of RNA editing events [34,35] or reflect the natural variation between individual plants and plant parts. The general error rate of the sequencing technique was noted to be very low, but isoenzymes coded by less common allelic variants would have been missed without manual sequence verification. Twenty-six of the resulting coding sequences, including allelic variants missed in the transcriptome sequencing and assembly processes, were codon optimized, synthesized and transformed to *P. pastoris* for expression.

This study reveals that for the coverage of all isoenzymes including allelic variants represented by the cDNA library sequenced, manual work to verify the resulting transcript sequences cannot be avoided. However, the allelic variants represent only a minor part of the newly discovered enzymes. The quality of the sequences is very high and differences to genomic DNA minimal, confirming that the enzyme discovery method described in this study for high-throughput applications would not necessarily require manual verification of the sequencing by laborious Sanger sequencing of amplified genomic DNA. However, manual curation of the contigs of interest and splitting of the data in contigs with clear assembly conflicts can be done and could be worthwhile, as especially the additive effects of amino acid changes in collapsed contigs could cause problematic changes in the enzyme structure. The primary enzyme discovery pipeline utilized in this study provides a functional approach to find proteins of interest for heterologous production, giving an example of an affordable standardized sequencing project. Despite the large amount of useful data produced by the NGS approach, our study showed that sequence confirmation and data validation should not be neglected.

For the heterologous secretory production of single isoenzymes in *P. pastoris*, the codon usages of the coding sequences were optimized for efficient translation, and fragments corresponding to the predicted mature isoenzymes were produced synthetically. When the signal peptide prediction with SignalP led to two alternative signal peptide junctions, the mature peptides corresponding to the longer signal peptide variants were ordered as synthetic fragments, and the signal peptide variants with shorter mature peptide were successfully amplified via PCR. Correct cloning of all genes into *P. pastoris* expression vectors was verified by Sanger sequencing. Since the used *P. pastoris* expression vector already contains the signal sequence of the *S. cerevisiae* mating factor α, the isoenzymes were produced without the predicted natural signal sequences. Twenty-two isoenzymes showed peroxidase activity with at least one of the substrates used. All activities were measured with four different assays over a pH range from 4.5 to 7. Interestingly, for each assay (Table 4) a different isoenzyme showed the highest activity thus suggesting variable substrate specificities or pH optima. This observation further emphasizes the importance of the availability of a large group of individually produced pure isoenzymes to be able to comprehensively respond to the need of variable performance parameters including substrate specificity, activity, stability, and operating pH optimum. Four of the isoenzymes did not show peroxidase activity with the assays used. This could either be due to very low yields of active enzyme, totally inactive enzyme or unsuitable assay conditions.

The isoenzyme C1A was reported to be the most abundant isoenzyme in *A. rusticana *[1] and was thus expected to be found in the transcriptome. However, only two raw reads covering a minor part of the coding sequence could be detected. This might either suggest over-normalization of the cDNA library decreasing the total counts of the putatively most abundant transcripts to almost zero [45], or happen due to naturally occurring genetic variation with phenotypic correlation to adaptations to natural environments ranging from pathogens, light conditions or abiotic stress to a variety of other environmental perturbations [8,46,47]. Although mRNA originating from all available plant parts was used to reach genes activated at diverse stages of *A. rusticana* growth, some developmental stages were not present and the absence of certain isoenzymes due to missing tissues cannot be ruled out. This finding might illustrate the high variance of *HRP* expression in *A. rusticana* plants and consequently the variance in the commercial HRP preparations, thus underlining the clear need for a reliable heterologous expression system that enables a consistent isoenzyme quality.

Peroxidase isoenzymes have been suggested to have multiple roles in the plant and thus also be variedly expressed depending on both biotic and abiotic factors [8]. To roughly estimate the expression levels of the newly discovered peroxidase genes, their GC contents and codon usages were assessed. Genes that are highly expressed have been suggested to possess a higher GC content and a more biased codon usage than genes with low expression levels [48]. A majority of both eukaryotic and prokaryotic species with large population sizes have been reported to have non-random codon usage mainly due to Darwinian selection between synonymous codons [49-51]. Highly expressed genes have been reported to use a restricted set of codons to ensure optimal translational efficiency [52,53]. In addition to gene expression levels, GC content has also been connected to gene regulation [54-57] and correlated with genomic features including methylation pattern [58], short intron length [59] and gene density [60] thus suggesting possible functional relevance. The codon usages and GC contents calculated using the verified coding sequences of the isoenzymes are described in Table 3. As expected, large variation between isoenzymes exist. These findings could suggest a spatial and temporal distribution of the isoenzymes in cellular processes [61].

Phylogenetic relationships of the HRP isoenzymes are shown in Figure 3. Whereas the previously known isoenzymes are closely related to each other, most of the new isoenzymes discovered in the transcriptome share higher evolutionary distance to the previously known HRPs. BLASTX analysis to the peroxidases of *A. thaliana* (Additional file 3) revealed that the *A. rusticana* peroxidases share 81% to 95% sequence similarity to the most similar isoenzyme of *A. thaliana*. Evolutionary distance does not necessarily correlate with altered substrate specificity, specific activity or optimal reaction conditions, but the discovery of new evolutionary branches with higher structural diversity does offer optimal conditions for the generation of an enzyme assortment with diverse properties for a wide variety of biomedical and industrial applications.

A combination of cDNA sequencing and gDNA verification in this study also provided valuable information of the intron-exon boundaries of the *HRP* genes. The number of exons in the isoenzymes was noted to vary from one to four, corresponding to zero to three introns. A large majority (75% of the peroxidase loci) of the isoenzymes were found to have four exons and three introns. Intron numbers have been reported to be highly conserved, but total intron length (total sum of the sizes of all introns within a gene) rather correlated to the GC-content of the gene [62]. Thus, intron number could be informative in terms of evolutionary origin and distance of the enzyme. In this study, intron numbers were found to correlate with the phylogenetic relationships of the amino acid sequences. Contigs with an unusual number of introns (none or two, Table 3) were situated in close proximity to each other furthest away from the previously known isoenzymes and clustered together when comparing the codon usage frequencies. With the information obtained from the reads, no alternative splicing could be observed.

The well-characterized isoenzyme HRP C1A has been reported to have a signal peptide consisting of 30 amino acids, and a carboxy-terminal extension suggested to target the protein to the vacuoles [63]. Also other known isoenzymes of the group C (C1B, C1C, C2, and C3) have been reported to have signal peptides varying in length from 9 amino acids (C1C) to 29 amino acids (C3). By observing the alignment of all previously known and newly discovered isoenzymes (Additional file 2), existence of signal sequences also in other previously known and most of the new isoenzymes seemed very probable. According to the signal sequence prediction (SignalP) performed, all isoenzymes seem to have a signal sequence varying in length from 18 to 31 amino acids (Table 3, Additional file 2). Isoenzyme C1C, previously reported to have a signal sequence of nine amino acids, was predicted to have - better corresponding to the sequences of the very closely related isoenzymes C1A and C1B - a signal sequence of 29 amino acids. In the case of unclear signal sequence prediction with more than one option for the length of the signal peptide, both forms were taken into consideration when planning the constructs for enzyme production in *P. pastoris*.

## Conclusions

To facilitate the possibilities for heterologous expression and isoenzyme characterization, we have elucidated the nucleotide sequences of 28 horseradish peroxidase isoenzymes by using the data obtained from *A. rusticana* 454 transcriptome sequence analysis with manual verification of PCR amplified genomic DNA. Although studies including transcriptome analysis of non-model species have become increasingly popular since the emergence of the NGS technologies, methods for the utilization of the 454 technology for the purpose of isoenzyme discovery in non-model plant species have not been established. In this project, transcriptome sequencing reads are further processed with alternative assemblies and manual sequence verification to determine the nucleotide sequences of all HRP isoenzymes. This study does not only contribute a set of transcripts, which can be used for marker development and genomic studies to understand agriculturally important traits in *A. rusticana*, but also provides valuable information of the peroxidase gene structure. Twenty-two of the verified isoenzymes have been produced in a form that was found active towards the tested substrates in *P. pastoris* utilizing a new *P. pastoris* expression platform [64], validating the success of the approach and providing first insights into the versatility of this large group of isoenzymes discovered.

## Methods

### Plant specimens, RNA extraction and quality analysis

Wild horseradish (*A. rusticana*) roots were purchased from local farmers and grown in the laboratory to obtain fresh roots, sprouts, stems and leaves. Tissues were collected in aliquots, frozen in liquid nitrogen and stored at -80°C. Total RNA from all available plant parts was isolated using RNaqueous kit (Applied Biosystems/Ambion, Austin, TX, USA) according to the manufacturer’s recommendations. Quality assessment to ensure RNA integrity was performed with Agilent 2100 Bioanalyzer (Agilent Technologies, Santa Clara, CA, USA).

### Normalized cDNA library construction and sequencing

Transcriptome sequence was obtained by a commercial service from LGC Genomics (Berlin, Germany). The methods used are roughly summarized as follows: mRNA was purified from total RNA using mRNA-ONLY™ Eukaryotic mRNA Isolation Kit (Epicentre, Madison, WI, USA). One μg of mRNA was used for first-strand cDNA synthesis and amplification according to the Mint-Universal cDNA Synthesis Kit user manual (Evrogen, Moscow, Russia), followed by a normalization reaction using the Trimmer Kit (Evrogen). Normalized material was re-amplified, digested (*Sfi*I), size-selected (>800 bp, LMP agarose), purified (Qiagen, Hilden, Germany) and ligated to pDNR-lib vector (Clontech, Saint-Germain-en-Laye, France) using the Fast Ligation Kit (New England Biolabs, Ipswich, MA, USA). The desalted ligation was used to transform NEB10b competent cells (New England Biolabs).

Roughly a million clones were plated on LB (lysogeny broth) + chloramphenicol (Cm) plates, scraped off the plates and stored as glycerol stocks at -70°C. Plasmid DNA was prepared using standard methods (Qiagen, Hilden, Germany), and digested with *Sfi*I. cDNA inserts were gel-purified (LMP-Agarose/MinElute Gel Extraction Kit, Qiagen) and ligated to high-molecular-weight DNA using a proprietary *Sfi*I-linker.

Library generation for the 454 FLX sequencing was carried out according to standard protocols (Roche/454 Life Sciences, Branford, CT 06405, USA). In short, the concatenated inserts were sheared randomly by nebulization to fragments ranging in size from 400 bp to 900 bp. These fragments were end polished and the 454 A and B adaptors that are required for the emulsion PCR and sequencing were ligated to the ends of the fragments. The resulting fragment library was sequenced on half a picotiterplate on the GS FLX using the Roche/454 Titanium chemistry. Sequence data can be accessed via the EMBL-EBI European Nucleotide Archive under the study accession number PRJEB5793.

### Assembly of the sequence reads to transcripts

Raw reads produced by the pyrosequencing process were screened for the *Sfi*I-linker that was used for concatenation and the linker sequences were clipped from the reads. Poly A/T sequences were mostly (~90%) removed with the linker. High-quality reads were selected using Newbler sequence filtering at default settings. The clipped, quality controlled reads were assembled into individual isotigs using the Roche/454 Newbler software (454 Life Sciences Corporation, version 2.5.3) with default settings (minimum read length 20, duplicate reads excluded, expected depth 0, seed step 12, seed length 16, seed count 1, minimum overlap length 40 bp, minimum overlap identity 90%, alignment identity score 2, alignment difference score -3).

### Discovery of peroxidases in the assembled contigs

The PSSM (position-specific scoring matrix) corresponding to known horseradish peroxidases was obtained from NCBI's Conserved Domain Database (cd00693, CDD v3.01) [65]. It was used in a tblastn search (e-value cutoff 1e-5) [66] on the assembled contigs to yield a preliminary set of *HRP* candidate sequences. This set was refined by filtering sequences whose translation mapped back to a domain different to the original profile PSSM in an rpsblast classification of the entire CDD or had a bit score lower than the NCBI-specified threshold for a specific domain match. The read composition of the refined set of contigs was manually reviewed using SeqMan (DNASTAR, Madison, Wisconsin, USA). Isotigs where two apparently different variants were assembled into one contig by the assembler were split. Protein coding regions were extended using read information if two or more reads contained the same sequence.

### Genome walking and manual verification of horseradish peroxidase sequences

The sequences of the identified peroxidase genes were manually verified by Sanger sequencing of PCR amplified genomic fragments using Phusion^TM^ High-Fidelity Polymerase (Finnzymes Oy, Espoo, Finland) and primers listed in Additional file 4. If no flanking regions were available for primer design, genome walking [67] was utilized to clarify and complete the sequences of the C– and N-termini. Therefore, 2 μg aliquots of genomic DNA were singly digested with *Bsp*143I, *Hin*dIII, *Psu*I (Fermentas, St. Leon-Rot, Germany), *Bsa*WI (New England Biolabs GmbH, Frankfurt am Main, Germany) or *Xho*II (Promega GmbH, Madison, WI, USA) in order to get fragments of 1 - 5 kb size. The digestion was stopped by heat inactivation of the enzymes, the fragmented DNA was precipitated with ethanol and the pellet was dissolved in 30 μL of distilled water. An adaptor was created by annealing adaptor strand 1 (5'-GTAATACGACTCACTATAGGGCACGCGTGGTCGACGGCCCGGGCTGGT-3') either to adaptor strand 2.a (3'-TCCCCGACCACTAG-5') for *Bsp*143I/*Psu*I-/*Xho*II-digested DNA, 2.b (3'-TCCCCGACCATTAA-5') for *Bsa*WI-digested DNA or 2.c (3'-TCCCCGACCATCGA-5') for *Hin*dIII- digested DNA.

In the annealing reaction, adaptor strand 1 was mixed in 1:1 molar ratio with adaptor strand 2.a/2.b/2.c (i.e. 13.7 μL of 100-μM adaptor strand 1 + 4.0 μL of 100 μM adaptor strand 2) and heated to 95 °C for 5 min. To anneal the two strands to a functional adaptor molecule, the mixture was allowed to slowly cool down to room temperature. The three differently annealed adaptors (1 + 2a, 1 + 2b, 1 + 2c) were ligated for three hours at room temperature with T4 DNA Ligase (Fermentas) to the digested DNA fragments, considering the specific 5' overhangs that have been created by the respective restriction enzymes. The ligation reaction was stopped by incubation for 5 min at 70°C and 70 μL of TE (Tris-EDTA) buffer was added. Two gene-specific primers and two adaptor primers were designed. The gene-specific primers were designed to bind approximately 100 bp from the end of the known sequence, considering that no restriction site of the restriction enzymes used for DNA digestion laid between the primer-binding site and the end of the known sequence. AdaptorPrimer1 (5'-GTAATACGACTCACTATAGGGC-3') and GeneSpecificPrimer1 were used as a primer pair for a first PCR with 1 μL of the DNA + adaptor ligation product as template DNA. One μL of the first PCR mix was used as template for a second PCR with AdaptorPrimer2 (5'-ACTATAGGGCACGCGTGGT-3') and GeneSpecificPrimer2. This second primer pair was designed to bind within the first PCR product. Both PCR steps were performed with an elongation time of 50 seconds.

A gene-specific DNA fragment as product from the second PCR was isolated from a preparative agarose gel, purified (SV DNA extraction kit, Promega) and sent to Sanger sequencing (LGC Genomics GmbH, Berlin, Germany), using AdaptorPrimer2 and the corresponding GeneSpecificPrimer2. If unspecific primer binding or nucleotide polymorphisms were suspected, the PCR products were cloned into the pJET 1.2blunt vector (GeneJet cloning kit, Promega), transformed to *E. coli* Top10 F' and plasmids from single colonies were isolated for sequencing to ensure the read consisted of only one allele.

The resulting gene sequences were submitted to EMBL [68] under accession numbers HE963800-HE963825 (Table 3).

### Codon usage, GC content, isoelectric point, signal sequence prediction, disulfide bridge prediction and phylogenetic analyses of the horseradish peroxidase isoenzymes

Codon usages and GC contents of the HRP isoenzymes were analyzed using CAIcal [69,70] and Mega5 [71,72]. The sequences of the *A. thaliana* Class III peroxidases were downloaded from TAIR [73]. Sequences were aligned with ClustalW2 [74,75]. The theoretical isoelectric points (pI) were calculated with ExPASy Compute pI/Mw tool [76]. Disulfide bridges were predicted with EDBCP tool [77-79]. Phylogenetic analyses were performed with CLC Main Workbench 6.6.2 (CLC bio, Aarhus Denmark) [80] and Mega5. The pylogentetic tree was generated with the "Create Tree" function of CLC Main Workbench using the UPGMA algorithm with a bootstrap analysis of 100 replicates, based on an alignment of the HRP amino acid sequences using the "Create Alignment" function with the following settings: Gap open cost: 10.0, Gap extension cost: 1.0, End gap cost: as any other, Alignment: Very accurate (slow). Signal sequences were predicted using SignalP 3.0 [81,82].

### Gene synthesis and heterologous expression in *Pichia pastoris*

The codon usages of 14 isoenzymes were optimized for the expression in *P. pastoris* using a novel algorithm (DNA2.0, Menlo Park, CA, USA, Mellitzer *et al.* manuscript in preparation). Further twelve isoenzymes including allelic variants were optimized using GeneDesigner 1.1.4.1 (DNA2.0) in accordance to the *P. pastoris* codon usage described by Abad *et al. *[83]. Signal sequence variants were generated by PCR amplification and all *HRP* genes were cloned into the shuttle vector pPpT4_alpha_S of a newly generated open source expression platform [64]. The vector pPpT4_alpha_S is a basic low-copy (1–5 copies/genome), zeocin™ resistance based expression vector for efficient secretory expression of heterologous proteins. Sanger sequencing of the plasmids verified successful cloning into the right frame. The linearized expression cassettes were transformed into *P. pastoris* wild-type CBS7435 based mut^s^ strain using standard protocols [84], and selected on zeocin™-containing plates. From each gene, 88 clones were picked to 96-well deep-well plates for cultivation and high-throughput screening of peroxidase activity. Two of the well expressing clones of each isoenzyme were streaked out to single colonies. Four single colonies of each clone were used for re-screening to estimate the reproducibility of the results. All media compositions and cultivation protocols used in this study were as previously described by [85]. Minimal media BMD1% (buffered minimal media with 1% dextrose) was supplemented with 5 mM ferrous sulfate heptahydrate (Sigma-Aldrich Handels Gmbh, Vienna, Austria) to ensure sufficient iron supply for heme biosynthesis.

### Peroxidase assays

ABTS (2,2’-azino-bis(3-ethylbenzthiazoline-6-sulfonic acid), TMB (3,3’,5’5-tetramethyl benzidine), pyrogallol (1,2,3-trihydroxybenzene) and guaiacol (2-methoxyphenol) assays were used to detect peroxidase activity essentially as described in [3,86,87]. ABTS assays were performed in 50 mM sodium acetate buffer pH 4.5 with 1 mM ABTS and 0.0026% (v/v) H_2_O_2._ For the TMB stock solution, TMB was dissolved in DMSO to a concentration of 4.16 mM. For the assay solution, TMB stock solution and 30% (v/v) H_2_O_2_ were diluted with 20 mM citrate buffer pH 5.5 to final concentrations of 0.416 mM and 0.006% (v/v), respectively. Guaiacol assays were performed in 10 mM sodium phosphate buffer pH 7.0 with 5 mM guaiacol and 0.0009% (v/v) H_2_O_2_. For the pyrogallol assay solution, pyrogallol (Sigma-Aldrich Handels Gmbh, Vienna, Austria) was dissolved in 10 mM potassium phosphate buffer pH6.0 containing 0.027% (v/v) H_2_O_2_ to a concentration of 45 mM. For all assays, 15 μl cultivation supernatant was mixed with 140 μl of the assay solution in a flat-bottom 96-well microtiterplate (Greiner Bio-One GmbH, Frickenhausen, Germany). The reaction kinetics were followed with Spectramax Plus^384^ spectrophotometer and SoftMax® Pro software (Molecular Devices, LLC) for 3–5 min at wavelengths 405 nm (ABTS), 650 nm (TMB), 470 nm (guaiacol) and 420 nm (pyrogallol). Enzyme activity was calculated using only time points fitting to linear increase of the absorbance (ΔmAU min^-1^).

### Availability of supporting data

*A. rusticana* transcriptome sequencing data is available via EMBL-EBI's European Nucleotide Archive (ENA) under the study accession number PRJEB5793 (http://www.ebi.ac.uk/ena/data/view/PRJEB5793). Nucleotide sequences of the novel identified HRPs have been deposited into ENA as well: accession numbers HE963800-HE963825 (http://www.ebi.ac.uk/ena/data/view/HE963800-HE963825). All other supporting data are included as additional files with this manuscript.

## Abbreviations

ABTS: 2,2’-azino-bis(3-ethylbenzthiazoline-6-sulfonic acid); AU: Absorption unit; BLAST: Basic local alignment search tool; BLASTX: Search protein databases using a translated nucleotide query; BMD: Buffered minimal media with dextrose; bp: Base pair; CDD: Conserved domains database; cDNA: Complementary DNA; DMSO: Dimethyl sulfoxide; EST: Expressed sequence tag; gDNA: Genomic DNA; HRP: Horseradish peroxidase; LB: Lysogenous broth; mRNA: messenger RNA; NGS: New generation sequencing; ORF: Open reading frame; PSSM: Position-specific scoring matrix; RIN: RNA integrity number; RSCU: Relative synonymous codon usage; SNV: Single nucleotide variation; tBLASTn: search translated nucleotide databases with a protein query; TE: tris-EDTA; TMB: 3,3’,5’5-tetramethyl benzidine.

## Competing interests

AG, FWK, and LN report a patent application (No. 11185833.8 - 2403), owned by Graz University of Technology, which relates to the novel recombinant horseradish peroxidase isoenzymes with improved technological properties described in this work and the authors do not have any objection on the patent right of authorship and ownership. All other authors declare that they have no competing interests.

## Authors’ contributions

AG, GGT and LN conceived the study and designed the experiments. LN collected the samples, isolated RNA, manually verified the read composition of the peroxidase contigs, and participated in the bioinformatic analysis. MS and GGT developed a pipeline to discover peroxidase isoenzymes in the contigs and participated in the bioinformatic analysis. LN and FWK verified the genomic sequences, planned the synthetic genes and performed protein expression in *Pichia pastoris*. AG initiated the project and contributed to the evaluation of the results. LN and GGT drafted the manuscript and all other authors revised it and approved the final version.

## Supplementary Material

Additional file 1 **Heatmaps of of the changes in the relative synonymous codon usages (ΔRSCU) of A) all the HRP isoenzymes verified in this study and B) the known *A. thaliana *peroxidases.** Each column represents one codon indicated along the bottom, each row one isoenzyme marked to the right side of the row. Isoenzymes are clustered by their codon usage similarity. Green cells correspond to underrepresented codons, red cells to overrepresented codons. Missing codons are marked with a grey cell. Click here for file

Additional file 2 Alignment of the amino acid sequences of the HRP isoenzymes. Click here for file

Additional file 3 **BLASTP of respective full length HRP amino acid sequence against non-redundant protein sequences (nr) database with *A. thaliana *(taxid:3702) as organism.** Click here for file

Additional file 4 Primers used in this study. Click here for file

## References

[B1] Veitch NC Horseradish peroxidase: a modern view of a classic enzyme Phytochemistry 2004 15 249 259 10.1016/j.phytochem.2003.10.022 14751298

[B2] Greco O Rossiter S Kanthou C Folkes LK Wardman P Tozer GM Dachs GU Horseradish peroxidase-mediated gene therapy: choice of prodrugs in oxic and anoxic tumor conditions Mol Cancer Ther 2001 15 151 160 12467232

[B3] Morawski B Quan S Arnold FH Functional expression and stabilization of horseradish peroxidase by directed evolution in *Saccharomyces cerevisiae* Biotechnol Bioeng 2001 15 99 107 10.1002/bit.1149 11505379

[B4] Wagner M Nicell JA Detoxification of phenolic solutions with horseradish peroxidase and hydrogen peroxide Water Res 2002 15 4041 4052 10.1016/S0043-1354(02)00133-1 12405413

[B5] Azevedo AM Martins VC Prazeres DMF Vojinović V Cabral JMS Fonseca LP Horseradish peroxidase: a valuable tool in biotechnology Biotechnol Annu Rev 2003 15 199 247 14650928 10.1016/s1387-2656(03)09003-3

[B6] Van de Velde F van Rantwijk F Sheldon RA Improving the catalytic performance of peroxidases in organic synthesis Trends Biotechnol 2001 15 73 80 10.1016/S0167-7799(00)01529-8 11164557

[B7] Hoyle MC High resolution of peroxidase-indoleacetic acid oxidase isoenzymes from horseradish by isoelectric focusing Plant Physiol 1977 15 787 793 10.1104/pp.60.5.787 16660185 PMC542715

[B8] Passardi F Cosio C Penel C Dunand C Peroxidases have more functions than a Swiss army knife Plant Cell Rep 2005 15 255 265 10.1007/s00299-005-0972-6 15856234

[B9] Fujiyama K Takemura H Shibayama S Kobayashi K Choi JK Shinmyo A Takano M Yamada Y Okada H Structure of the horseradish peroxidase isozyme C genes Eur J Biochem 1988 15 681 687 10.1111/j.1432-1033.1988.tb14052.x 3371352

[B10] Fujiyama K Takemura H Shinmyo A Okada H Takano M Genomic DNA structure of two new horseradish-peroxidase-encoding genes Gene 1990 15 163 169 10.1016/0378-1119(90)90002-9 2373366

[B11] Bartonek-Roxå E Eriksson H Mattiasson B The cDNA sequence of a neutral horseradish peroxidase Biochim Biophys Acta 1991 15 245 250 10.1016/0167-4781(91)90060-Y 2001399

[B12] Nielsen KL Indiani C Henriksen A Feis A Becucci M Gajhede M Smulevich G Welinder KG Differential Activity and Structure of Highly Similar Peroxidases. Spectroscopic, Crystallographic, and Enzymatic Analyses of Lignifying Arabidopsis thaliana Peroxidase A2 and Horseradish Peroxidase A2 Biochemistry 2001 15 11013 11021 10.1021/bi010661o 11551197

[B13] Morita Y Mikami B Yamashita H Lee JY Aibara S Sato M Katsube Y Tanaka N Lobarzewski J, Greppin H, Penel C, Gaspar T Primary and crystal structures of horseradish peroxidase isozyme E5 Biochemical, Molecular and Physiolgical Aspects of Plant Peroxidase 1991 Lublin and Geneva: University of M. Curie-Sklodowska and University of Geneva 81 88

[B14] Shannon LM Kay E Jow Y Shannon LXI Lew JY Chemistry and Metabolism of Macromolecules: Peroxidase Isozymes from Horseradish Roots: I. Isolation and physical Properties J Biol Chem 1966 15 2166 2172 5946638

[B15] Aibara S Yamashita H Mori E Kato M Morita Y Isolation and characterization of five neutral isoenzymes of horseradish peroxidase J Biochem 1982 15 531 539 7130156 10.1093/oxfordjournals.jbchem.a133961

[B16] Aibara S Kobayashi T Morita Y Isolation and properties of basic isoenzymes of horseradish peroxidase J Biochem 1981 15 489 496 7298597 10.1093/oxfordjournals.jbchem.a133496

[B17] Weber APM Weber KL Carr K Wilkerson C Ohlrogge JB Sampling the *Arabidopsis* transcriptome with massively parallel pyrosequencing Plant Physiol 2007 15 32 42 10.1104/pp.107.096677 17351049 PMC1913805

[B18] Parchman TL Geist KS Grahnen JA Benkman CW Buerkle CA Transcriptome sequencing in an ecologically important tree species: assembly, annotation, and marker discovery BMC Genomics 2010 15 180 10.1186/1471-2164-11-180 20233449 PMC2851599

[B19] Sun C Li Y Wu Q Luo H Sun Y Song J Lui EMK Chen S *De novo* sequencing and analysis of the American ginseng root transcriptome using a GS FLX Titanium platform to discover putative genes involved in ginsenoside biosynthesis BMC Genomics 2010 15 262 10.1186/1471-2164-11-262 20416102 PMC2873478

[B20] Schmid J Müller-Hagen D Bekel T Funk L Stahl U Sieber V Meyer V Transcriptome sequencing and comparative transcriptome analysis of the scleroglucan producer *Sclerotium rolfsii* BMC Genomics 2010 15 329 10.1186/1471-2164-11-329 20504312 PMC2887420

[B21] Wong MML Cannon CH Wickneswari R Identification of lignin genes and regulatory sequences involved in secondary cell wall formation in *Acacia auriculiformis* and *Acacia mangium* via *de novo* transcriptome sequencing BMC Genomics 2011 15 342 10.1186/1471-2164-12-342 21729267 PMC3161972

[B22] Brown AP Kroon JTM Swarbreck D Febrer M Larson TR Graham I a Caccamo M Slabas AR Tissue-specific whole transcriptome sequencing in castor, directed at understanding triacylglycerol lipid biosynthetic pathways PLoS One 2012 15 e30100 10.1371/journal.pone.0030100 22319559 PMC3272049

[B23] Clark SM Vaitheeswaran V Ambrose SJ Purves RW Page JE Transcriptome analysis of bitter acid biosynthesis and precursor pathways in hop (*Humulus lupulus*) BMC Plant Biol 2013 15 12 10.1186/1471-2229-13-12 23347725 PMC3564914

[B24] Egan AN Schlueter J Spooner DM Applications of next-generation sequencing in plant biology Am J Bot 2012 15 175 185 10.3732/ajb.1200020 22312116

[B25] Johnson MTJ Carpenter EJ Tian Z Bruskiewich R Burris JN Carrigan CT Chase MW Clarke ND Covshoff S Depamphilis CW Edger PP Goh F Graham S Greiner S Hibberd JM Jordon-Thaden I Kutchan TM Leebens-Mack J Melkonian M Miles N Myburg H Patterson J Pires JC Ralph P Rolf M Sage RF Soltis D Soltis P Stevenson D Stewart CN Evaluating methods for isolating total RNA and predicting the success of sequencing phylogenetically diverse plant transcriptomes PLoS One 2012 15 e50226 10.1371/journal.pone.0050226 23185583 PMC3504007

[B26] Schliesky S Gowik U Weber APM Bräutigam A RNA-Seq Assembly - Are We There Yet? Front 2012 15 220 23056003 10.3389/fpls.2012.00220 PMC3457010

[B27] The 1KP Project http://www.onekp.com

[B28] Xiao M Zhang Y Chen X Lee E-J Barber CJS Chakrabarty R Desgagné-Penix I Haslam TM Kim Y-B Liu E MacNevin G Masada-Atsumi S Reed DW Stout JM Zerbe P Zhang Y Bohlmann J Covello PS De Luca V Page JE Ro DK Martin VJJ Facchini PJ Sensen CW Transcriptome analysis based on next-generation sequencing of non-model plants producing specialized metabolites of biotechnological interest J Biotechnol 2013 15 122 134 10.1016/j.jbiotec.2013.04.004 23602801

[B29] The PhytoMetaSyn Project http://www.phytometasyn.ca/

[B30] Góngora-Castillo E Childs KL Fedewa G Hamilton JP Liscombe DK Magallanes-Lundback M Mandadi KK Nims E Runguphan W Vaillancourt B Varbanova-Herde M Dellapenna D McKnight TD O’Connor S Buell CR Development of transcriptomic resources for interrogating the biosynthesis of monoterpene indole alkaloids in medicinal plant species PLoS One 2012 15 e52506 10.1371/journal.pone.0052506 23300689 PMC3530497

[B31] The Medicinal Plant Genomics Resource http://www.medicinalplantgenomics.msu.edu

[B32] Wang T-Y Chen H-L Lu M-YJ Chen Y-C Sung H-M Mao C-T Cho H-Y Ke H-M Hwa T-Y Ruan S-K Hung K-Y Chen C-K Li J-Y Wu Y-C Chen Y-H Chou S-P Tsai Y-W Chu T-C Shih C-C a Li W-H Shih M-C Functional characterization of cellulases identified from the cow rumen fungus *Neocallimastix patriciarum W5* by transcriptomic and secretomic analyses Biotechnol. Biofuels 2011 15 24 10.1186/1754-6834-4-24 21849025 PMC3177772

[B33] Marchler-Bauer A Panchenko AR Shoemaker BA Thiessen PA Geer LY Bryant SH CDD: a database of conserved domain alignments with links to domain three-dimensional structure Nucleic Acids Res 2002 15 281 283 10.1093/nar/30.1.281 11752315 PMC99109

[B34] Shah SP Morin RD Khattra J Prentice L Pugh T Burleigh A Delaney A Gelmon K Guliany R Senz J Steidl C Holt R a Jones S Sun M Leung G Moore R Severson T Taylor G a Teschendorff AE Tse K Turashvili G Varhol R Warren RL Watson P Zhao Y Caldas C Huntsman D Hirst M Marra M a Aparicio S Mutational evolution in a lobular breast tumour profiled at single nucleotide resolution Nature 2009 15 809 813 10.1038/nature08489 19812674

[B35] Chepelev I Wei G Tang Q Zhao K Detection of single nucleotide variations in expressed exons of the human genome using RNA-Seq Nucleic Acids Res 2009 15 e106 10.1093/nar/gkp507 19528076 PMC2760790

[B36] Garg R Patel RK Tyagi AK Jain M *De novo* assembly of chickpea transcriptome using short reads for gene discovery and marker identification DNA Res 2011 15 53 63 10.1093/dnares/dsq028 21217129 PMC3041503

[B37] Codon Usage Database http://www.kazusa.or.jp/codon/cgi-bin/showcodon.cgi?species=3704

[B38] Cirulli ET Singh A Shianna KV Ge D Smith JP Maia JM Heinzen EL Goedert JJ Goldstein DB Screening the human exome: a comparison of whole genome and whole transcriptome sequencing Genome Biol 2010 15 R57 10.1186/gb-2010-11-5-r57 20598109 PMC2898068

[B39] Toth AL Varala K Newman TC Miguez FE Hutchison SK Willoughby DA Simons JF Egholm M Hunt JH Hudson ME Robinson GE Wasp gene expression supports an evolutionary link between maternal behavior and eusociality Science 2007 15 441 444 10.1126/science.1146647 17901299

[B40] Vera JC Wheat CW Fescemyer HW Frilander MJ Crawford DL Hanski I Marden JH Rapid transcriptome characterization for a nonmodel organism using 454 pyrosequencing Mol Ecol 2008 15 1636 1647 10.1111/j.1365-294X.2008.03666.x 18266620

[B41] Novaes E Drost DR Farmerie WG Pappas GJ Grattapaglia D Sederoff RR Kirst M High-throughput gene and SNP discovery in *Eucalyptus grandis*, an uncharacterized genome BMC Genomics 2008 15 312 10.1186/1471-2164-9-312 18590545 PMC2483731

[B42] Pop M Salzberg SL Bioinformatics challenges of new sequencing technology Trends Genet 2008 15 142 149 10.1016/j.tig.2007.12.006 18262676 PMC2680276

[B43] Natarajan P Parani M *De novo* assembly and transcriptome analysis of five major tissues of *Jatropha curcas L*. using GS FLX titanium platform of 454 pyrosequencing BMC Genomics 2011 15 191 10.1186/1471-2164-12-191 21492485 PMC3087711

[B44] Huse SM Huber JA Morrison HG Sogin ML Welch DM Accuracy and quality of massively parallel DNA pyrosequencing Genome Biol 2007 15 R143 10.1186/gb-2007-8-7-r143 17659080 PMC2323236

[B45] Hale MC McCormick CR Jackson JR Dewoody JA Next-generation pyrosequencing of gonad transcriptomes in the polyploid lake sturgeon (*Acipenser fulvescens*): the relative merits of normalization and rarefaction in gene discovery BMC Genomics 2009 15 203 10.1186/1471-2164-10-203 19402907 PMC2688523

[B46] Delker C Pöschl Y Raschke A Ullrich K Ettingshausen S Hauptmann V Grosse I Quint M Natural variation of transcriptional auxin response networks in *Arabidopsis thaliana* Plant Cell 2010 15 2184 2200 10.1105/tpc.110.073957 20622145 PMC2929100

[B47] Alonso-Blanco C Aarts MGM Bentsink L Keurentjes JJB Reymond M Vreugdenhil D Koornneef M What has natural variation taught us about plant development, physiology, and adaptation? Plant Cell 2009 15 1877 1896 10.1105/tpc.109.068114 19574434 PMC2729614

[B48] Wright F The “effective number of codons” used in a gene Gene 1990 15 23 29 10.1016/0378-1119(90)90491-9 2110097

[B49] Gouy M Gautier C Codon usage in bacteria: correlation with gene expressivity Nucleic Acids Res 1982 15 7055 7074 10.1093/nar/10.22.7055 6760125 PMC326988

[B50] Ikemura T Codon usage and tRNA content in unicellular and multicellular organisms Mol Biol Evol 1985 15 13 34 3916708 10.1093/oxfordjournals.molbev.a040335

[B51] Sharp PM Matassi G Codon usage and genome evolution Curr Opin Genet Dev 1994 15 851 860 10.1016/0959-437X(94)90070-1 7888755

[B52] Bulmer M The Selection-Mutation-Drift Theory of Synonymous Codon Usage Genetics 1991 15 897 907 1752426 10.1093/genetics/129.3.897 PMC1204756

[B53] Li WH Models of nearly neutral mutations with particular implications for nonrandom usage of synonymous codons J Mol Evol 1987 15 337 345 10.1007/BF02134132 3110426

[B54] Carels N Bernardi G The compositional organization and the expression of the *Arabidopsis* genome FEBS Lett 2000 15 302 306 10.1016/S0014-5793(00)01476-9 10788631

[B55] Carels N Bernardi G Two classes of genes in plants Genetics 2000 15 1819 1825 10747072 10.1093/genetics/154.4.1819 PMC1461008

[B56] Vinogradov AE DNA helix: the importance of being GC-rich Nucleic Acids Res 1838–1844 15 31 10.1093/nar/gkg296 PMC152811 12654999

[B57] Zhang L Kasif S Cantor CR Broude NE GC/AT-content spikes as genomic punctuation marks Proc Natl Acad Sci U S A 2004 15 16855 16860 10.1073/pnas.0407821101 15548610 PMC534751

[B58] Jabbari K Bernardi G CpG doublets, CpG islands and Alu repeats in long human DNA sequences from different isochore families Gene 1998 15 123 127 10.1016/S0378-1119(98)00474-0 9931467

[B59] Galtier N Piganeau G D. M Duret L GC-Content Evolution in Mammalian Genomes: The Biased Gene Conversion Hypothesis Genetics 2001 15 907 911 11693127 10.1093/genetics/159.2.907 PMC1461818

[B60] Mouchiroud D D’Onofrio G Aïssani B Macaya G Gautier C Bernardi G The distribution of genes in the human genome Gene 1991 15 181 187 2055469 10.1016/0378-1119(91)90364-h

[B61] Cosio C Dunand C Specific functions of individual class III peroxidase genes J Exp Bot 2009 15 391 408 10.1093/jxb/ern318 19088338

[B62] Rayko E Jabbari K Bernardi G The evolution of introns in human duplicated genes Gene 2006 15 41 47 16356663 10.1016/j.gene.2005.09.038

[B63] Welinder KG Covalent structure of the glycoprotein horseradish peroxidase (EC 1.11.1.7) FEBS Lett 1976 15 19 23 10.1016/0014-5793(76)80804-6 1001465

[B64] Näätsaari L Mistlberger B Ruth C Hajek T Hartner FS Glieder A Deletion of the *Pichia pastoris KU70* homologue facilitates platform strain generation for gene expression and synthetic biology PLoS One 2012 15 e39720 10.1371/journal.pone.0039720 22768112 PMC3387205

[B65] Marchler-Bauer A Lu S Anderson JB Chitsaz F Derbyshire MK DeWeese-Scott C Fong JH Geer LY Geer RC Gonzales NR Gwadz M Hurwitz DI Jackson JD Ke Z Lanczycki CJ Lu F Marchler GH Mullokandov M Omelchenko MV Robertson CL Song JS Thanki N Yamashita RA Zhang D Zhang N Zheng C Bryant SH CDD: a Conserved Domain Database for the functional annotation of proteins Nucleic Acids Res 2011 15 D225 D229 10.1093/nar/gkq1189 21109532 PMC3013737

[B66] Camacho C Coulouris G Avagyan V Ma N Papadopoulos J Bealer K Madden TL BLAST+: architecture and applications BMC Bioinf 2009 15 421 10.1186/1471-2105-10-421 PMC2803857 20003500

[B67] Shyamala V Ferro-Luzzi Ames G Genome walking by single-specific-primer Gene 1989 15 1 8 10.1016/0378-1119(89)90132-7 2691331

[B68] The European Molecular Biology Laboratory (EMBL) http://www.ebi.ac.uk/embl/

[B69] Puigbò P Bravo IG Garcia-Vallve S CAIcal: a combined set of tools to assess codon usage adaptation Biol Direct 2008 15 38 10.1186/1745-6150-3-38 18796141 PMC2553769

[B70] CAIcal Server http://genomes.urv.es/CAIcal/

[B71] Tamura K Peterson D Peterson N Stecher G Nei M Kumar S MEGA5: molecular evolutionary genetics analysis using maximum likelihood, evolutionary distance, and maximum parsimony methods Mol Biol Evol 2011 15 2731 2739 10.1093/molbev/msr121 21546353 PMC3203626

[B72] Molecular Evolutionary Genetics Analysis (MEGA) software http://www.megasoftware.net/

[B73] The Arabidopsis Information Resource (TAIR) http://www.arabidopsis.org/

[B74] Higgins DG Thompson JD Gibson TJ Using CLUSTAL for multiple sequence alignments Methods Enzym 1996 15 383 402 10.1016/s0076-6879(96)66024-8 8743695

[B75] CLUSTALW2 http://www.ebi.ac.uk/Tools/msa/clustalw2/

[B76] ExPASy Compute pI/Mw tool http://web.expasy.org/compute_pi/

[B77] Cheng J Saigo H Baldi P Large-scale prediction of disulphide bridges using kernel methods, two-dimensional recursive neural networks, and weighted graph matching Proteins 2006 15 617 629 16320312 10.1002/prot.20787

[B78] Lippi M Passerini A Punta M Rost B Frasconi P MetalDetector: a web server for predicting metal-binding sites and disulfide bridges in proteins from sequence Bioinformatics 2008 15 2094 2095 10.1093/bioinformatics/btn371 18635571 PMC2732205

[B79] Ensemble-based Disulfide Bonding Connectivity Pattern prediction server http://biomedical.ctust.edu.tw/edbcp/

[B80] CLC bio: CLC Main Workbench 6.6.2 http://www.clcbio.com

[B81] Bendtsen JD Nielsen H von Heijne G Brunak S Improved prediction of signal peptides: SignalP 3.0 J Mol Biol 2004 15 783 795 10.1016/j.jmb.2004.05.028 15223320

[B82] SignalP 3.0 Server http://www.cbs.dtu.dk/services/SignalP/

[B83] Abad S Kitz K Hörmann A Schreiner U Hartner FS Glieder A Real-time PCR-based determination of gene copy numbers in *Pichia pastoris* Biotechnol J 2010 15 413 420 10.1002/biot.200900233 20349461

[B84] Lin-Cereghino J Wong WW Xiong S Giang W Luong LT Vu J Johnson SD Lin-Cereghino GP Condensed protocol for competent cell preparation and transformation of the methylotrophic yeast *Pichia pastoris* Biotechniques 2005 15 44 48 10.2144/05381BM04 15679083 PMC2504082

[B85] Weis R Luiten R Skranc W Schwab H Wubbolts M Glieder A Reliable high-throughput screening with *Pichia pastoris* by limiting yeast cell death phenomena FEMS Yeast Res 2004 15 179 189 10.1016/j.femsyr.2004.06.016 15489201

[B86] Ryan BJ O’Fágáin C Arginine-to-lysine substitutions influence recombinant horseradish peroxidase stability and immobilisation effectiveness BMC Biotechnol 2007 15 86 10.1186/1472-6750-7-86 18053254 PMC2234406

[B87] Morawski B Lin Z Cirino P Joo H Bandara G Arnold FH Functional expression of horseradish peroxidase in *Saccharomyces cerevisiae* and *Pichia pastoris* Protein Eng 2000 15 377 384 10.1093/protein/13.5.377 10835112

