# Supplementary material for: Peroxidase gene discovery from the horseradish transcriptome
Source: BMC Genomics. 2014 Mar 24;15:227. doi: 10.1186/1471-2164-15-227 (PMC3987668; doi:10.1186/1471-2164-15-227)
Supplement: Additional file 1 — Heatmaps of of the changes in the relative synonymous codon usages (ΔRSCU) of A) all the HRP isoenzymes verified in this study and B) the known A. thaliana peroxidases. Each column represents one codon indicated along the bottom, each row one isoenzyme marked to the right side of the row. Isoenzymes are clustered by their codon usage similarity. Green cells correspond to underrepresented codons, red cells to overrepresented codons. Missing codons are marked with a grey cell. [file 1471-2164-15-227-S1.pdf]

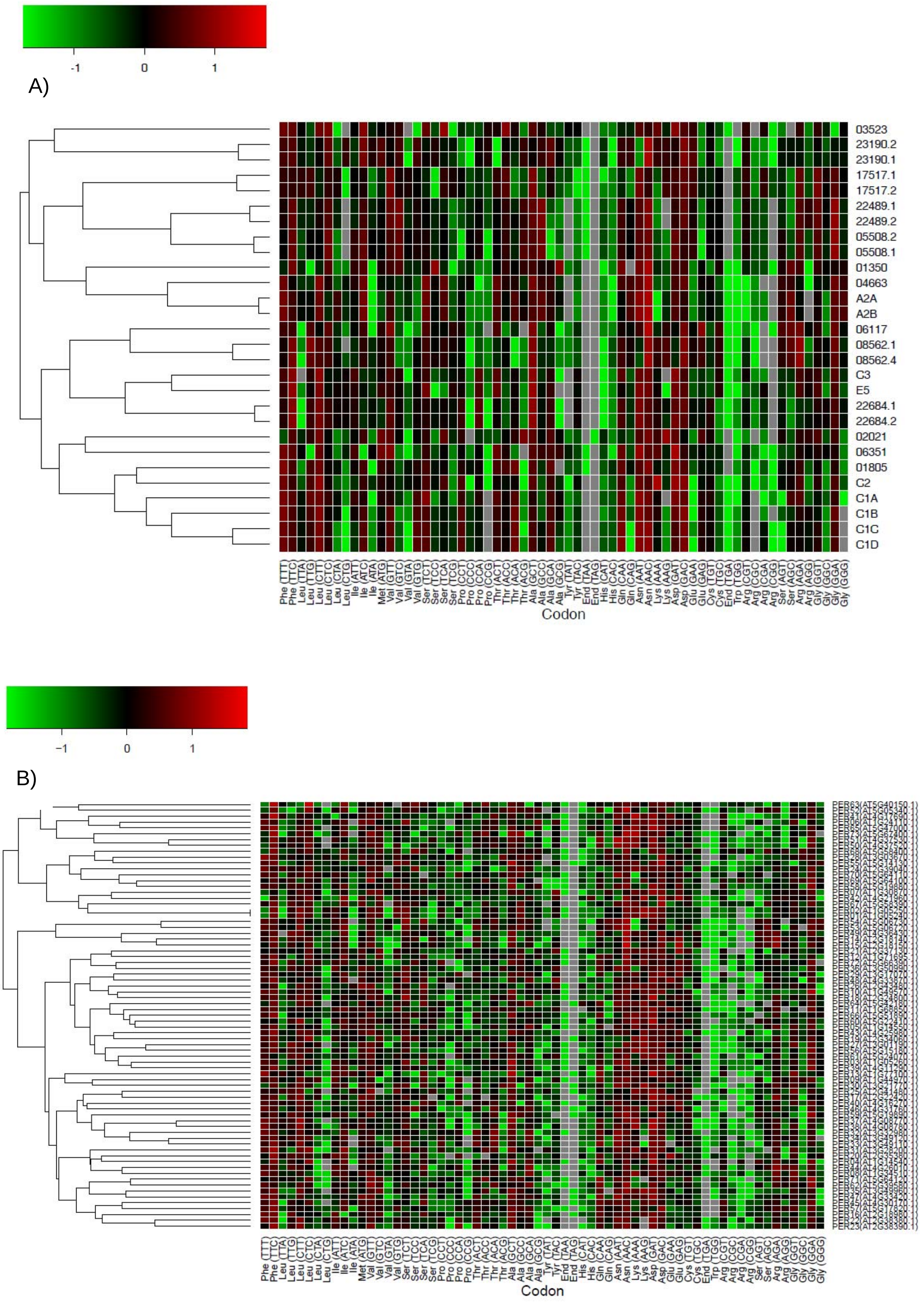

Additional file 1: Heat maps of the changes in the relative synonymous codon usages (ΔRSCU) of A) all the HRP isoenzymes verified in this study and B) the known *A. thaliana* peroxidases. Each column represents one codon indicated along the bottom, each row one isoenzyme marked to the right side of the row. Isoenzymes are clustered by their codon usage similarity. Green cells correspond to underrepresented codons, red cells to overrepresented codons. Missing codons are marked with a grey cell.
