# Supplementary material for: Peroxidase gene discovery from the horseradish transcriptome
Source: BMC Genomics. 2014 Mar 24;15:227. doi: 10.1186/1471-2164-15-227 (PMC3987668; doi:10.1186/1471-2164-15-227)
Supplement: Additional file 2 — Alignment of the amino acid sequences of the HRP isoenzymes. [file 1471-2164-15-227-S2.pdf]

Additional file 2. Alignment of the amino acid sequences of the HRP isoenzymes

```
C1C_gDNA          ---MH--SPSSTSFTWATLITLGCLMLHASFS--------NAQLTPTFYDNSCPNVSNIV 47
C1D_gDNA          ---MH--SPSSTSFTWATLITLGCLMLHASFS--------NAQLTPTFYDNSCPNVSNIV 47
C1B_gDNA          ---MH--SPSSTSFTWI-LITLGCLAFYASLS--------DAQLTPTFYDTSCPNVSNIV 46
C1A_gDNA          ---MHF-SSSSTLFTCITLIPLVCLILHASLS--------DAQLTPTFYDNSCPNVSNIV 48
01805_gDNA        ---MHFSTSSSSLSTWTTLITLGCLMLHSFKS--------SAQLTPTFYDSTCPSVFSIV 49
C2_gDNA           ---MH---SSSSLIKLG----FLLLLLNVSLS--------HAQLSPSFYDKTCPQVFDIA 42
C3_gDNA           ---MG--FSPLISCSAMGALILSCLLLQASNS--------NAQLRPDFYFRTCPSVFNII 47
E5_gDNA           ---MV--VSPFFSCSAMGALILGCLLLQASN----------AQLRPDFYSRTCPSVFNII 45
22684.1_gDNA      ---MG--FSPSFSSSSIGVLILGCLLLQASNS--------NAKLRPDFYLKTCPSVFQII 47
22684.2_gDNA      ---MG--FSPSFSSSSIGVLILGCLLLQASNS--------NAKLRPDFYLKTCPSVFQII 47
A2A_gDNA          ---MA---VTNLSTTCDGLFIISLLVIVSSLFGT-----SSAQLNATFYSGTCPNASAIV 49
A2B_gDNA          ---MA---VTNLSTTCDGLFIISLLVIVSSLFGT-----SSAQLNATFYSGTCPNASAIV 49
04663_gDNA        ---MA---ATSSSTTCDGLFIISLLVIASSLFGT-----SSAQLNATFYSGTCPNASAIV 49
08562.4_gDNA      ---MAR--LTSILLLLSLLCFFPLCLCDKS--YG-----G--KLFPGFYAHSCPQAGEIV 46
08562.1_gDNA      ---MAR--LTSILLLLSLLCFFPLCLCDKS--YG-----G--KLFPGFYAHSCPQAGEIV 46
06117_gDNA        ---MAR--IGSFLVVISLACVLTLCICDDESNYG-----GQGKLFPGFYSSSCPKAEEIV 50
01350_gDNA        ---MAS--NQRISILVLVVTFLVQGNYNNV---------VEAQLTPNFYSTSCPNLLSTV 46
23190.1_gDNA      MAMSYSIRVLTFLMLISLMAVTLNLLSTAEAKKPRRDVPIVKGLSWNFYQRACPKVEKII 60
23190.2_gDNA      MAMSYSIRVLTFLMLISLMAVTLNLLSTAEAKKPRRDVPIVKGLSWNFYQRACPKVEKII 60
03523_gDNA        ---MAELKSLSLILLFTLLT------TTIESR-----------LTTNFYSKSCPRFFDIV 40
06351_gDNA        ---MVRANLVSVILLMHVIVG-----FPFHARG----------LSMTYYMMSCPMAEQIV 42
05508.1_gDNA      --------MGLIRSLCVFITFLSCIISSAHGQAISIS---IT-IRIGFYLTTCPTAEIIV 48
05508.2_gDNA      --------MGLIRSLCVFITFLSCIISSAHGQAISIS---IT-IRIGFYLTTCPTAEIIV 48
22489.1           --------MEFVRSLCVFITFLGCLISSAHGQAAARRPGPISGTRIGFYLTTCPTAEIIV 52
22489.2           --------MEFVRSLCVFITFLGCLISSAHGQAAARRPGPISGTRIGFYLTTCPTAEIIV 52
17517.1_gDNA      --------MGRGYNLLLILVTFLVLVAAVTARR----------PRVGFYGNRCRKVESIV 42
17517.2_gDNA      --------MGRGYNLLLILVTFLVLVAAVTARR----------PRVGFYGNRCRKVESIV 42
02021_gDNA        ---MRT--MKRLNVAVAVAVTATVLMGMLGSSE--------AQLQMNFYAKSCPNAEKII 47
                                                                 :*   *

C1C_gDNA          RDIIINELRSDPRIAASILRLHFHDCFVNGCDASILLDNTTSFRTEKDAFGNANSAR-GF 106
C1D_gDNA          RDIIINELRSDPRIAASILRLHFHDCFVNGCDASILLDNTTSFRTEKDAFGNANSAR-GF 106
C1B_gDNA          RDIIINELRSDPRITASILRLHFHDCFVNGCDASILLDNTTSFLTEKDALGNANSAR-GF 105
C1A_gDNA          RDTIVNELRSDPRIAASILRLHFHDCFVNGCDASILLDNTTSFRTEKDAFGNANSAR-GF 107
01805_gDNA        RDTIVNELRSDPRIAASILRLHFHDCFVNGCDASILLDNTTSFRTEKDAAPNANSAR-GF 108
C2_gDNA           TNTIKTALRSDPRIAASILRLHFHDCFVNGCDASILLDNTTSFRTEKDAFGNARSAR-GF 101
C3_gDNA           GDIIVDELRTDPRIAASLLRLHFHDCFVRGCDASILLDNSTSFRTEKDAAPNANSAR-GF 106
E5_gDNA           KNVIVDELQTDPRIAASILRLHFHDCFVRGCDASILLDTSKSFRTEKDAAPNVNSAR-GF 104
22684.1_gDNA      GNVIVDELQSDPRIAASLLRLHFHDCFVRGCDASVLLDNSTSFQSEKDAAPNANSAR-GF 106
22684.2_gDNA      GNVIVDELQSDPRIAASLLRLHFHDCFVRGCDASVLLDNSTSFQSEKDAAPNANSAR-GF 106
A2A_gDNA          RSTIQQAFQSDTRIGASLIRLHFHDCFVNGCDASILLDDSGSIQSEKNAGPNANSAR-GF 108
A2B_gDNA          RSTIQQAFQSDTRIGASLIRLHFHDCFVNGCDASILLDDSGSIQSEKNAGPNANSAR-GF 108
04663_gDNA        RSTIQQALQSDPRIGASLIRLHFHDCFVNGCDGSLLLDDTGSIQSEKNAPANANSAR-GF 108
08562.4_gDNA      RSVVAKAVARETRMAASLMRLHFHDCFVQGCDGSLLLDSSGRIVSEKGSNPNSRSAR-GF 105
08562.1_gDNA      RSVVAKAVARETRMAASLMRLHFHDCFVQGCDGSLLLDSSGKIVSEKGSNPNSRSAR-GF 105
06117_gDNA        RSVVAKAVARETRMAASLMRLHFHDCFVQGCDGSLLLDSSGSIVTEKNSNPNSRSAR-GF 109
01350_gDNA        QSAVKSAVNSEARMGASIVRLFFHDCFVNGCDGSILLDDTSSFTGEQNANPNRNSAR-GF 105
23190.1_gDNA      KKELKKVFKRDIGLAAAILRIHFHDCFVQGCEASVLLAGSASGPGEQSSIPNLTLRQQAF 120
23190.2_gDNA      KKELKKVFKRDIGLAAAILRIHFHDCFVQGCEASVLLAGSASGPGEQSSIPNLTLRQQAF 120
03523_gDNA        RDTISNKQITTPTTAAATIRLFFHDCFPNGCDASILISSTAFNTAERDSSINLSLPGDGF 100
06351_gDNA        KNSVNNALQADPTLAAGLIRMLFHDCFIEGCDASILLDSTKDNTAEKDSPANLSLRG--Y 100
05508.1_gDNA      RNAVRAGFNSDPRIAPGILRMHFHDCFVQGCDGSVLISGS---NTERTAVPNLSLRG--F 103
05508.2_gDNA      RNAVRAGFNSDPRIAPGILRMHFHDCFVQGCDGSVLISGS---NTERTAVPNLSLRG--F 103
22489.1           RNAVRAGFNSDPRIAPGILRMHFHDCFVLGCDGSVLISGS---NTERTAVPNLNLRG--F 107
22489.2           RNAVRAGFNSDPRIAPGILRMHFHDCFVLGCDGSVLISGS---NTERTAVPNLNLRG--F 107
17517.1_gDNA      RSVVRSHFRCNPANAPGILRMYFHDCFVNGCDGSILLAGN---TSERTAGPNRSLRG--F 97
17517.2_gDNA      RSVVRSHFRCNPANAPGILRMHFHDCFVNGCDGSILLAGN---TSERTAGPNRSLRG--F 97
02021_gDNA        SDHIQKHIPSGPSLAAPLIRMHFHDCFVRGCDGSVLINSTSG-NAEKDSAPNLTLRG--F 104
                   . :           .  :*: *****  **:.*:*:  .     *: :  *       :

C1C_gDNA          PVVDRIKAAVERACPRTVSCADVLTIAAQQSVNLAGGPSWRVPLGRRDSRQAFLDLANAN 166
C1D_gDNA          PVVDRIKAAVERACPRTVSCADVLTIAAQQSVNLAGGPSWRVPLGRRDSRQAFLDLANTN 166
C1B_gDNA          PTVDRIKAAVERACPRTVSCADVLTIAAQQSVNLAGGPSWRVPLGRRDSLQAFLDLANAN 165
C1A_gDNA          PVIDRMKAAVESACPRTVSCADLLTIAAQQSVTLAGGPSWRVPLGRRDSLQAFLDLANAN 167
01805_gDNA        PVIDTMKAAVERACPRTVSCADLLTIAAQQSVNLAGGPSWRVPLGRRDSVQAFFDLANTN 168
C2_gDNA           DVIDTMKAAVEKACPKTVSCADLLAIAAQKSVVLAGGPSWKVPSGRRDSLRGFMDLANDN 161
C3_gDNA           GVIDRMKTSLERACPRTVSCADVLTIASQISVLLSGGPWWPVPLGRRDSVEAFFDLANTA 166
E5_gDNA           NVIDRMKTALERACPRTVSCADILTIASQISVLLSGGPSWAVPLGRRDSVEAFFDLANTA 164
22684.1_gDNA      DVVDRMKAALEKACPGTVSCADVLAISAQISVLLSGGPWWPVLLGRRDGVEAFFDLANTA 166
22684.2_gDNA      DVVDRMKAALEKACPGTVSCADVLAISAQISVLLSGGPWWPVLLGRRDGVEAFFDLANTA 166
A2A_gDNA          NVVDNIKTALENTCPGVVSCSDILALASEASVSLTGGPSWTVLLGRRDSLTANLAGANSA 168
```

```
A2B_gDNA          NVVDNIKTALENTCPGVVSCSDILALASEASVSLTGGPSWTVLLGRRDSLTANLAGANSA 168
04663_gDNA        NVVDDIKTALENACPGIVSCSDILALASEASVSLAGGPSWTVLVGRRDGLTANLSGANSS 168
08562.4_gDNA      DVVDQIKAELEKQCPGTVSCADALTLAARDSSVLTGGPSWVVSLGRRDSRSASLSGSNNN 165
08562.1_gDNA      DVVDQIKAELEKQCPGTVSCADALTLAARDSSVLTGGPSWVVSLGRRDSRSASLSGSNNN 165
06117_gDNA        EVVDEIKAALENECPNTVSCADALTLAARDSSVLTGGPSWMVPLGRRDSTSASLSGSNNN 169
01350_gDNA        NVIDNIKAAVEKACPGVVSCADILAIAARDSVVVLGGPNWTVKVGRRDARTASQAAANSN 165
23190.1_gDNA      VVINNLRALVQKQCGQVVSCSDILALAARDSIVLSGGPDYAVPLGRRDSLAFATPETTLA 180
23190.2_gDNA      VVINNLRALVQKQCGQVVSCSDILALAARDSIVLSGGPDYAVPLGRRDSLAFATPETTLA 180
03523_gDNA        DVIVRAKTAIELACPNTVSCSDIITVATRDLLVTVGGPYYDVYLGRRDSRISKSSLLTDL 160
06351_gDNA        EIIDDAKEKVENMCPGVVSCADIVAMAARDAVFWAGGPYYDIPKGRFDGKRSK-IEDTRN 159
05508.1_gDNA      EVIENAKTQLEATCPGVVSCADILALAARDTVVLTRGIGWQVPTGRRDGRVS-VASNANN 162
05508.2_gDNA      EVIENAKTQLEAACPGVVSCADILALAARDTVVLTRGIGWQVPTGRRDGRVS-VASNANN 162
22489.1           EVIDNAKTQLEATCPGVVSCADILALAARDTVVLTRGLGWQVPTGRRDGRVS-VASNANN 166
22489.2           EVIDNAKTQLEATCPGVVSCADILALAARDTVVLTRGLGWQVPTGRRDGRVS-VASNANN 166
17517.1_gDNA      EAIEEAKTRLENACPNTVSCADILTLAARDAVVWTGGKGWSVPLGRLDGRRS-EASDVN- 155
17517.2_gDNA      EAIEEAKTRLENACPNTVSCADILTLAARDAVVWTGGKGWSVPLGRLDGRRS-EASDVN- 155
02021_gDNA        GFVERIKTLLEAECPKTVSCADIIALTARDAVVATGGPSWKVPTGRRDGRISNTTEALNN 164
                    :   :  ::  *   ***:* :::::.       *  : :  ** *.

C1C_gDNA          -LPAPSFTLPELKAAFANVGLNRPSDLVALSGGHTFGKNQCRFIMDRLYNFSNTGLPDPT 225
C1D_gDNA          -LPAPSFTLPQLKAAFANVGLNRPSDLVALSGGHTFGKNQCRFIMDRLYNFSNTGLPDPT 225
C1B_gDNA          -LPAPFFTLPQLKDAFAKVGLDRPSDLVALSGGHTFGKNQCRFIMDRLYNFSNTGLPDPT 224
C1A_gDNA          -LPAPFFTLPQLKDSFRNVGLNRSSDLVALSGGHTFGKNQCRFIMDRLYNFSNTGLPDPT 226
01805_gDNA        -LPAPFFTLPQLKASFSNVGLDRPEDLVALSGGHTFGKNQCQFIMDRLYNFSNTGLPDPT 227
C2_gDNA           -LPGPSSTLQVLKDKFRNVGLDRPSDLVALSGGHTFGKNQCQFIMDRLYNFSNSGKPDPT 220
C3_gDNA           -LPSPFFTLAQLKKAFADVGLNRPSDLVALSGGHTFGRAQCQFVTPRLYNFNGTNRPDPT 225
E5_gDNA           -LPSPFFTLAQLKKAFADVGLNRPSDLVALSGGHTFGRARCLFVTARLYNFNGTNRPDPT 223
22684.1_gDNA      -LPNPFAPLTELKEKFADVGLKRASDLVALSGAHTFGRAQCLLVTPRLYNFSGTNKPDPT 225
22684.2_gDNA      -LPNPFAPLTELKEKFADVGLKRASDLVALSGAHTFGRAQCLLVTPRLYNFSGTNKPDPT 225
A2A_gDNA          -IPSPFEGLSNITSKFSAVGLN-TNDLVALSGAHTFGRARCGVFNNRLFNFSGTGNPDPT 226
A2B_gDNA          -IPSPFEGLSNITSKFSAVGLN-TNDLVALSGAHTFGRARCGVFNNRLFNFSGTGNPDPT 226
04663_gDNA        -LPSPFEGLNNITSKFLAVGLN-TTDVVVLSGAHTFGRGQCVTFNNRLFNFNGTGSPDPT 226
08562.4_gDNA      -IPAPNNTFQTILSKFNRQGLD-VTDLVALSGSHTIGFSRCTSFRQRLYNQSGNGRPDMT 223
08562.1_gDNA      -IPAPNNTFQTILSKFNRQGLD-VTDLVALSGSHTIGFSRCTSFRQRLYNQSGNGRPDMT 223
06117_gDNA        -IPAPNNTFNTILSRFNSQGLD-LTNVVALSGSHTIGFSRCTSFRQRLYNQSGNGSPDTT 227
01350_gDNA        -IPAPTSSLSQLISSFSAVGLS-TRDMVALSGAHTIGQSRCTSFRTRIYN-------ETN 216
23190.1_gDNA      NLPPPFANASQLISDFNDRNLN-ITDLVALSGGHTIGIAHCPSFTDRLYPNQ-----DPT 234
23190.2_gDNA      NLPPPFANASQLISDFNDRNLN-ITDLVALSGGHTIGIAHCPSFTDRLYPNQ-----DPT 234
03523_gDNA        -LPLPSSPISKTIRQFESKGFT-IQEMVALSGAHSIGFSHCKEFVNRVAGN------NTG 212
06351_gDNA        -LPSPFLNASQLIQTFGNRGFS-PQDVVALSGAHTLGVARCSSFKARLTTP------DSS 211
05508.1_gDNA      -LPGPRDSVAVQQQKFSALGLN-TRDLVVLAGGHTLGTAGCGVFRDRLFNN-----TDPN 215
05508.2_gDNA      -LPGPRDSVAVQQQKFSALGLN-TRDLVVLAGGHTLGTAGCGVFRDRLFNN-----TDPN 215
22489.1           -LPGPRDSVAVQQQKFSAVGLN-TRDLVVLAGGHTIGTAGCGVFRDRLFNN-----TDPN 219
22489.2           -LPGPRDSVAVQQQKFSAVGLN-TRDLVVLAGGHTIGTAGCGVFRDRLFNN-----TDPN 219
17517.1_gDNA      -LPGPSDPVAKQKQDFAAKNLN-TLDLVTLVGGHTIGTAGCGLVRGRFFNFNGTGQPDPS 213
17517.2_gDNA      -LPGPSDPVAKQKQDFAAKNLN-TLDLVTLVGGHTIGTAGCGLVRGRFFNFNGTGQPDPS 213
02021_gDNA        -IPPPTSNFTTLQRLFANQGLN-LKDLVLLSGAHTIGVSHCSSMNTRLYNFSTTVKQDPS 222
                   :* *          *   .:    ::* * *.*::*   *  .  *.         :

C1C_gDNA          LNTTYLQTLRQ-QCPRNGN--QSVLVDFDLRTPTVFDNKYYVNLKEQKGLIQSDQELFSS 282
C1D_gDNA          LNTTYLQTLRQ-QCPRNGN--QSVLVDFDLRTPTVFDNKYYVNLKEQKGLIQSDQELFSS 282
C1B_gDNA          LNTTYLQTLRQ-QCPLNGN--QSVLVDFDLRTPTVFDNKYYVNLKEQKGLIQSDQELFSS 281
C1A_gDNA          LNTTYLQTLRG-LCPLNGN--LSALVDFDLRTPTIFDNKYYVNLEEQKGLIQSDQELFSS 283
01805_gDNA        LNTTYLQTLRV-QCPRNGN--QSVLVDFDLRTPTVFDNKYYVNLKEHKGLIQTDQELFSS 284
C2_gDNA           LDKSYLSTLRK-QCPRNGN--LSVLVDFDLRTPTIFDNKYYVNLKENKGLIQSDQELFSS 277
C3_gDNA           LDPTYLVQLRA-LCPQNGN--GTVLVNFDVVTPNTFDRQYYTNLRNGKGLIQSDQELFST 282
E5_gDNA           LNPSYLADLRR-LCPRNGN--GTVLVNFDVMTPNTFDNQFYTNLRNGKGLIQSDQELFST 280
22684.1_gDNA      LNPSYLVELRR-LCPQNGN--GTVLLNFDLVTPNAFDRQYYTNLRNGKGLIQSDQELFST 282
22684.2_gDNA      LNPSYLVELRR-LCPQNGN--GTVLLNFDLVTPNAFDRQYYTNLRNGKGLIQSDQELFST 282
A2A_gDNA          LNSTLLSSLQQ-LCPQNGS--ASTITNLDLSTPDAFDNNYFANLQSNNGLLQSDQELFST 283
A2B_gDNA          LNSTLLSSLQQ-LCPQNGS--ASTITNLDLSTPDAFDNNYFANLQSNNGLLQSDQELFST 283
04663_gDNA        LNSTLLSSLQQ-ICPQNGS--GSAITNLDLTTPDAFDSNYYTNLQSNNGLLQSDQELFSN 283
08562.4_gDNA      LEQSFAANLRQ-RCPRSGG--DQILSVLDIISAAKFDNSYFKNLIENKGLLNSDQVLFNS 280
08562.1_gDNA      LEQSFAANLRQ-RCPRSGG--DQILSVLDIISAAKFDNSYFKNLIENKGLLNSDQVLFSS 280
06117_gDNA        LEQSYAANLRH-RCPRSGG--DQNLSELDINSAGRFDNSYFKNLIENMGLLNSDQVLFSS 284
01350_gDNA        INAAFATTRQR-TCPRTSGSGDGNLAPLDVTTAASFDNNYFKNLMTQRGLLHSDQELFNG 275
23190.1_gDNA      MNKSFANSLKR-TCP--TAN-SSNTQVNDIRSPDVFDNKYYVDLMNRQGLFTSDQDLFVD 290
23190.2_gDNA      MNKSFANSLKR-TCP--TAN-SSNTQVNDIRSPDVFDNKYYVDLMNRQGLFTSDQDLFVD 290
03523_gDNA        YNPRFAQALKQ-ACSNYPKD-PTLSVFNDIMTPNRFDNMYYQNIPKGLGLLESDHGLYSD 270
06351_gDNA        LDSTFANTLTR-TCN--AGD-NAEQPFD--ATRNDFDNAYFNALQRKSGVLFSDQTLFNT 265
05508.1_gDNA      VDQPFLTQLQT-KCPRNGD--GSVRVDLDTGSGTTFDNSYFINLSRGRGVLESDHVLWTD 272
05508.2_gDNA      VDQPFLTQLQT-KCPRNGD--GSVRVDLDTGSGTTFDNSYFINLSRGRGVLESDHVLWTD 272
22489.1           VNQLFLTQLQT-QCPQNGD--GAVRVDLDTGSGTTFDNSYFINLSRGRGVLESDHVLWTD 276
```

```
22489.2           VNQLFLTQLQT-QCPQNGD--GSVRVDLDTGSGTTFDNSYFINLSRGRGVLESDHVLWTD 276
17517.1_gDNA      IDPSFVPLVQA-RCPQNGN--ATTRVDLDTGSAGDFDTSYLSNVRSSRVVLQSDLVLWKD 270
17517.2_gDNA      IDPSFVPLVQA-RCPQNGN--ATTRVDLDTGSAGDFDTSYLSNVRSSRVVLQSDLVLWKD 270
02021_gDNA        LDSEYAANLKANKCKSLND--NTTILEMDPGSSKTFDLSYYRLVLKRRGLFQSDSALTTN 280
                   :           *                 :   **  :   :     :: :*  *

C1C_gDNA          PNATDTIPLVRSYADGTQ---TFFNAFVEAMNRMGNITPLTG-TQGEIRLNCRVVNSNSL 338
C1D_gDNA          PNATDTIPLVRSYADGTQ---TFFNAFVEAMNRMGNITPLTG-TQGEIRLNCRVVNSNSL 338
C1B_gDNA          PNATDTIPLVRSFADGTQ---KFFNAFVEAMNRMGNITPLTG-TQGEIRLNCRVVNSNSL 337
C1A_gDNA          PNATDTIPLVRSFANSTQ---TFFNAFVEAMDRMGNITPLTG-TQGQIRLNCRVVNSNSL 339
01805_gDNA        PNAADTIPLVRSYADGTQ---KFFNAFMEAMNRMGNITPLTG-TQGQIRQNCRVINSNSL 340
C2_gDNA           PDASDTIPLVRAYADGQG---KFFDAFVEAMIRMGNLSPSTG-KQGEIRLNCRVVNSKPK 333
C3_gDNA           P-GADTIPLVNLYSSNTF---AFFGAFVDAMIRMGNLRPLTG-TQGEIRQNCRVVNSR-- 335
E5_gDNA           P-GADTIPLVNLYSSNTL---SFFGAFADAMIRMGNLRPLTG-TQGEIRQNCRVVNSR-- 333
22684.1_gDNA      P-GADTIPLVNLYSKNTF---AFFGAFVDAIIRMGNIQPLTG-TQGEIRQNCRVVNSR-- 335
22684.2_gDNA      P-GADTIPLVNLYSKNTF---AFFGAFVDAIIRMGNIQPLTG-TQGEIRQNCRVVNSR-- 335
A2A_gDNA          T-GSATIAVVTSFASNQT---LFFQAFAQSMINMGNISPLTG-SNGEIRLDCKKVNGS-- 336
A2B_gDNA          T-GSATITVVTSFASNQT---LFFQAFAQSMINMGNISPLTG-SNGEIRLDCKKVNGS-- 336
04663_gDNA        T-GSPTIAIVNSFASNQT---LFFEAFAQSMIKMGNISPLTG-TSGEIRQDCKAVNGQSS 338
08562.4_gDNA      N--EKSRELVKKYAEDQG---EFFEQFAESMIKMGNISPLTG-SSGEIRKNCRKINS--- 331
08562.1_gDNA      N--EKSRELVKKYAEDQG---EFFEQFAESMIKMGNISPLTG-SSGEIRKNCRKINS--- 331
06117_gDNA        N--DESRELVKKYAEDQE---EFFEQFAESMVKMGNISPLTG-SSGQIRKNCRKINS--- 335
01350_gDNA        G--S-TDSIVRGYSNNPS---SFSSDFAAAMIKMGDISPLTG-SSGEIRKVCGRTN---- 324
23190.1_gDNA      K---RTRGIVESFAIDQN---LFFDHFTVAMIKMGQMSVLTG-TQGEIRSNCSARNTASF 343
23190.2_gDNA      K---RTRGIVESFAIDQN---LFFDHFTVAMIKMGQMSVLTG-TQGEIRSNCSARNTASF 343
03523_gDNA        P---RTRPFVDLYARDQD---LFFKDFARAMQKLSLFGVKTG-RRGEIRRRCDAIN---- 319
06351_gDNA        P---RTRNLVNGYALNQA---KFFFDFQQAMRKMSNLDVKLG-SQGEIRQNCRTIN---- 314
05508.1_gDNA      P---ATRPIVQQLMSSSG---NFNAEFARSMVKMSNIGVVTG-TNGEIRKVCSAIN---- 321
05508.2_gDNA      P---ATRPIVQQLMSSSG---NFNAEFARSMVKMSNIGVVTG-TNGEIRKVCSAIN---- 321
22489.1           P---ATRPIVQQLMSPRG---NFNAEFARSMVRMSNIGVVTG-ANGEIRRVCSAVN---- 325
22489.2           P---ATRPIVQQLMSPRG---NFNAEFARSMVRMSNIGVVTG-ANGEIRRVCSAVN---- 325
17517.1_gDNA      T---ETRAIIERLLGLRRPVLRFGSEFGKSMTKMSLIEVKTRLSDGEIRRVCSAIN---- 323
17517.2_gDNA      T---ETRAIIERLLGLRRPVLRFGSEFGKSMTKMSLIEVKTRLSDGEIRRVCSAIN---- 323
02021_gDNA        S---ATLKMINDLVNGPEK--KFLKAFAKSMEKMGRVKVKTG-SAGVIRTRCSVAGS--- 331
                       :  .:            *   *  :: .:. .        * **  *   .

C1C_gDNA          LHDIVEVVDFVSSM------ 352
C1D_gDNA          LHDIVEVVDFVSSM------ 352
C1B_gDNA          LHDIVEVVDFVSSM------ 351
C1A_gDNA          LHDMVEVVDFVSSM------ 353
01805_gDNA        LHDIVEIVDFVSSM------ 354
C2_gDNA           IMDVVDTNDFASSI------ 347
C3_gDNA           IRGMENDDGVVSSI------ 349
E5_gDNA           IRGMENDDGVVSSM------ 347
22684.1_gDNA      IKGMENDGGVVSSI------ 349
22684.2_gDNA      IRGMENDDGVVSSI------ 349
A2A_gDNA          --------------------
A2B_gDNA          --------------------
04663_gDNA        ATKAEDIQMQSDGPVSLADM 358
08562.4_gDNA      --------------------
08562.1_gDNA      --------------------
06117_gDNA        --------------------
01350_gDNA        --------------------
23190.1_gDNA      ISVLEEGIVEEALSMI---- 359
23190.2_gDNA      ISVLVEGIVEEALSMI---- 359
03523_gDNA        --------------------
06351_gDNA        --------------------
05508.1_gDNA      --------------------
05508.2_gDNA      --------------------
22489.1           --------------------
22489.2           --------------------
17517.1_gDNA      --------------------
17517.2_gDNA      --------------------
02021_gDNA        --------------------
```
