# Supplementary material for: Peroxidase gene discovery from the horseradish transcriptome
Source: BMC Genomics. 2014 Mar 24;15:227. doi: 10.1186/1471-2164-15-227 (PMC3987668; doi:10.1186/1471-2164-15-227)
Supplement: Additional file 3 — BLASTP of respective full length HRP amino acid sequence against non-redundant protein sequences (nr) database with A. thaliana (taxid:3702) as organism. [file 1471-2164-15-227-S3.pdf]

Additional file 3. BLASTP of respective full length HRP amino acid sequence against non-redundant protein sequences (nr) database with Arabidopsis thaliana (taxid:3702) as organism.

#	HRP	*A. thaliana* gene accession #	*A. thaliana* peroxidase (database)	identities	positives	max score	total score	query coverage %	E value	max identity %
1	C1A	NP_190481.1	peroxidase 34	320/353	338/353	649	649	100	0	91
2	C1B_15901	NP_190480.1	peroxidase 33	306/342	320/342	635	635	97	0	89
3	C1C_25148	NP_190480.1	peroxidase 33	303/332	317/332	631	631	100	0	91
4	C1D_25148	NP_190480.1	peroxidase 33	303/332	316/332	630	630	100	0	91
5	C2_04627	NP_192617.1	peroxidase 37	297/332	310/332	610	610	95	0	89
6	C3	NP_181373.1	peroxidase 23	313/349	327/349	644	644	100	0	90
7	A2A	NP_196290.1	peroxidase 53	318/336	327/336	635	635	100	0	95
8	A2B	NP_196290.1	peroxidase 53	317/336	326/336	636	636	100	0	95
9	E5	NP_181372.1	peroxidase 22	298/349	314/349	597	597	100	0	85
10	1805	NP_850652.1	peroxidase 32	326/349	337/349	677	677	98	0	93
11	22684.1	NP_181372.1	peroxidase 22	291/349	318/349	607	607	100	0	83
12	22684.2	NP_181372.1	peroxidase 22	289/349	317/349	603	603	100	0	83
13	1350	NP_196153.1	peroxidase 52	304/324	312/324	600	600	100	0	94
14	2021	NP_188814.1	peroxidase 30	300/331	312/331	613	613	100	0	91
15	23190.1	NP_177313.1	peroxidase 12	327/359	340/359	613	613	99	0	91
16	23190.2	NP_177313.1	peroxidase 12	327/359	340/359	674	674	100	0	91
17	4663	NP_196291.1	peroxidase 54	267/292	278/292	481	481	98	6E-175	91
18	6351	NP_567919.1	peroxidase 47	298/314	308/314	610	610	99	0	95
19	3523	NP_189460.1	peroxidase 31	266/299	288/299	495	495	93	1E-180	89
20	5508.1	NP_201217.1	peroxidase 71	267/329	295/329	538	538	99	0	81
21	5508.2	NP_201217.1	peroxidase 71	265/328	293/328	544	544	100	0	80
22	22489.1	NP_201217.1	peroxidase 71	279/329	298/329	563	563	99	0	85
23	22489.2	NP_201217.1	peroxidase 71	280/329	298/329	565	565	99	0	85
24	6117	NP_179407.1	peroxidase 15	311/337	327/337	533	533	99	0	92
25	17517.1	NP_201215.1	peroxidase 69	275/334	288/334	539	539	100	0	82
26	17517.2	NP_201215.1	peroxidase 69	276/334	288/334	541	541	100	0	83
27	8562.1	NP_195361.1	peroxidase 49	311/331	320/331	592	592	99	0	94
28	8562.4	NP_195361.1	peroxidase 49	311/331	320/331	592	592	99	0	94
