# Supplementary material for: Peroxidase gene discovery from the horseradish transcriptome
Source: BMC Genomics. 2014 Mar 24;15:227. doi: 10.1186/1471-2164-15-227 (PMC3987668; doi:10.1186/1471-2164-15-227)
Supplement: Additional file 4 — Primers used in this study. [file 1471-2164-15-227-S4.pdf]

Additional file 4. Primers used for the verification of HRP sequences from *A. rusticana*

Number	Name	Sequence
P10-729	01350fw1	GGATGCGATGGTTCGATTTTAC
P10-727	01350fw2	GCGACACCGACAAAACAATTG
P10-728	01350rv1	GAGCGGAATTGCGGTTTG
P10-726	01350rv2	TGCGCGCCTGAAAAAAATGAAC
P10-725	06117fw1	GAGATTTAACAGCCAAGGTCTC
P10-724	06117rv1	CTCTTGAGAACTTAAGGAAGCG
P10-723	08562.1.4fw1	GAGCTGGGTGGTTTCATTAG
P10-722	08562.1.4rv1	GCTTTAGCTACGACTGATCTC
P10-721	08562.1.4rv2	CAAAAACCACACCGAGTTAGC
P10-720	17517fw1	CTAGTTACGTTTTTAGTATTGGTCG
P10-719	17517rv1	GACCAAATATGTAACTATTTGATTAATATACATTG
P10-573	A2_Nterm_Strepfw1	GTCCCACCCACAGTTCGAGAAGTCTGCACAATTGAATGCAACTTTCTATTCCG
P10-574	A2_Nterm_Strepfw2	ATATCTCGAGAAGAGAGAGGCCGAAGCTTGGTCCCACCCACAGTTCGAGAAG
P10-374	A2ex1rv	CGAAAAAGATCAAGAAGCCTTTATG
P10-381	A2ex4fw	CTTTGCTCAGTCCATGATCAAC
P10-382	A2ex4rv	GCCATTATGTGTTTTTTGAAAAACAAGAG
P10-375	A2in1ex2fw	CACGACTGCTTTGTTAATGTATAC
P10-376	A2in1ex2rv	GCTCTGGATGCTTCCAC
P10-377	A2in2fwa	CCGTGTCTTTGGTAATTAGTAATTAC
P10-379	A2in2fwb	GTTATATATATATAATTTGTCTTATAAATTATGTTTTAGTAATAATATAG
P10-378	A2in2rva	CTCCTGTCTGAAGATGATCAAAC
P10-380	A2in2rvb	GACTATCTCGTCTTCCTAATAATAC
P10-395	B1ex1fw	CTTAAACCAATAAAAGATAAGTTTCCTCTTAC
P10-396	B1ex1rv	GCGTATCCACAAAATCATCAATTAAAC
P10-397	B1ex2fw	GGTAGTAATTAAAGATTTGATTATGATTTCAAC
P10-398	B1ex2rv	GATAAAATTAAAACTAAAAAAAAGAGATAAATTAATGTTGC
P10-436	B2CtermSpecific1a	GTAAACCTATACAGCAAGAACACG
P10-438	B2CtermSpecific2	CTTCGGTGCATTCGTTGACG
P10-399	B2ex1fw	TAGTCTGTCTTCCTCTTGAAAAAAG
P10-400	B2ex1rv	CATGTTTTTTTCTTTTTCGGATAAAAAGATAAG
P10-403	B2ex2fw	GAGGCTAAAAGCAATTTTTAATTAATAAATACAATC
P10-404	B2ex2rv	ATAATATATTTTGATTAGTTTCACCCGATATAAG
P10-408	B2ex3rv	ATAATACATAGAGTAGGTTATATAGATTGAAAC
P10-411	B2ex4fw	TAAACATATATACAATGTATCTAAACTTTACTTTTTTTTG
P10-412	B2ex4rv	CGTCGTTCTCCATACCCT
P10-401	B2in1fw	CTCGTATTGCAGCTAGTCTC
P10-402	B2in1rv	GCATCTTTCTCGGACTGAAATG
P10-405	B2in2fw	CCTTGAGAAAGCTTGTCCTG
P10-406	B2in2rv	CTCTCCTCCCCAACAAAAC
P10-409	B2in3fw	GCGCCTCAGATCTAGTTG
P10-410	B2in3rv	GTCACAAGTAGACATTGTGCTC
P10-329	C1Aex3in3fw	GAGATAGCTTACAAGCATTTCTG
P10-330	C1Aex3in3rv	CCCTAACAAAAGAAAGAGAGATC
P10-331	C1Aex4fw	CACCATTTGATATAGTTGTATTTAGTGAG
P10-332	C1Aex4rv	GTAGCCACATATGGCGTC

Number	Name	Sequence
P10-325	C1Ain1fw	GTAAATTACTACTTTTCATATTTCTATTTCGTTAC
P10-325	C1Ain1fw	GTAAATTACTACTTTTCATATTTCTATTTCGTTAC
P10-326	C1Ain1rv	GCGTCACAACCCTTTCAAAAATC
P10-326	C1Ain1rv	GCGTCACAACCCTTTCAAAAATC
P10-327	C1Ain2fw	CGCAGATTTGCTCACCATTG
P10-328	C1Ain2rv	CCGCCTATAATAATAACCATTTTGTTTTG
P10-333	C1Bin1fw	GTTTTATCCTTTAGATATTGATAAATCACCTC
P10-335	C1Bin2fw	GAAATGATGTTGTGTTGTCTAACAATATC
P10-336	C1Bin2rv	GTATGCTCCATTCATTACAAACATTG
P10-337	C1Bin3fw	GACCTCCTGCCTATAATATAATAG
P10-338	C1Bin3rv	GATGCCTTTGCAAAAGTTGGC
P10-339	C1Cex1fw	GCTTCATGCATCTTTTTCCAATG
P10-340	C1Cex1rv	CACAACGCAATAGAAATATGAAAAGTAATATC
P10-343	C1Cex2fw	GTTACCGGTGGTAAAGATTTAGC
P10-344	C1Cex2rv	GAGGAATTGATATATTAAAATTTAAAACAATGATTTTAAC
P10-347	C1Cex3fw	ACAACGATAGCCTAAGTTTGAAAAG
P10-348	C1Cex3rv	ATTAAACTATACCAAATGGTGTTTAGTTTTCT
P10-348	C1Cex3rv	ATTAAACTATACCAAATGGTGTTTAGTTTTCT
P10-341	C1Cin1fw	CATTATCAATGAGTTACGATCGG
P10-342	C1Cin1rv	GCCTTGATTCTGTCAACCACA
P10-345	C1Cin2fw	CATGCCCAAGAACTGTTTCATG
P10-345	C1Cin2fw	CATGCCCAAGAACTGTTTCATG
P10-350	C1Cin3ex4rv	GCAGATCGAAATCCACCAAG
P10-351	C2ex1fw	CAAACCTAACCAAAGAATTTTATCTTAGAG
P10-352	C2ex1rv	CAGGTTTTTTACTAGCGTTAATAAAAAGTC
P10-353	C2in1ex2fwa	CACTAGATTCAATATTTTCACGTATATATTAATTAAG
P10-355	C2in1ex2fwb	CAAAAGAGTTGAAATATACTAAAAATATAATCTTACTAG
P10-357	C2in1ex2fwc	CAAAAGAAAACACCGGAGTTAAATAAAAATAG
P10-354	C2in1ex2rva	ATCAATCATATCCGAAAACGACAAC
P10-356	C2in1ex2rvb	GCGATTGGTCCATTTATAAATACTG
P10-358	C2in1ex2rvc	CCATCCTATCCTGAGCTATC
P10-360	C2in1ex2rvd	TCCTAAATGATGTAGTGTTGTCTAG
P10-361	C2in2fw	CTTTTACAATGCTATGCATCTATACATC
P10-362	C2in2rv	CGCCTATTTTATTTTCATTTTCAAGTAAACAAT
P10-365	C2in3ex4fwb	CAACAAATACTATGTGAATCTCAAAGAG
P10-364	C2in3ex4rva	CTCGGACCAAAGGGATAG
P10-366	C2in3ex4rvb	GTAGTAAAGAAAAAGATAACATTGATCATTTATTATTAG
P10-549	contig01350fw	CAACTCCAACTCCAAGTCTATTC
P10-550	contig01350rv	GATCAAATCTTATTCTCACCGAATC
P10-551	contig02021fw	AAGAGTATTGAGAAACAAGATCGAG
P10-552	contig02021rv	AACAGAAAACATCACTTTCCGAC
P10-545	contig04791fw	TCTCACTTTCTCTCTTCCGC
P10-546	contig04791rv	CATATAGATTTATGTGTTTTAACATTAACCAAAAAC
P10-543	contig06117fw	CTTCTTCTTTCTTCTGGATCTAG
P10-544	contig06117rv	CCCAAAACATCTCTCATTTTTATTTCG
P10-555	contig08562fw	GTAATGGCAAGACTCACTAGC

Number	Name	Sequence
P10-556	contig08562rv	CCCAATACTTTCCTCATTTCAAGA
P10-547	contig17517fw	CAACAACAACTTTTACAAAGCTCAAAG
P10-548	contig17517rv	CTATAGCAAGCTTATTCCATAAAATAAGTG
P10-439	E5CtermSpecific1	CAGCAGCAACACGTTATCG
P10-440	E5CtermSpecific2	CGTTCTTCGGAGCATTCGC
P10-383	E5ex1fw	CTCAAATCATAGTCTATCATCCTC
P10-384	E5ex1rv	GTCATGGTTTTTTTTTATTAATAATAAAAACATAAGTTAAG
P10-387	E5ex2fw	CAAGTACAATCGTCATATAACGTATAATATC
P10-388	E5ex2rv	GACGTCAAAATTTCATAACATATTTTTATTAATTTCAC
P10-393	E5ex4fw	GAATTATAAGATAAGATGGTAAAACGACAAAAC
P10-394	E5ex4rv	CCCATCCTAATCATTGCATCAG
P10-385	E5in1fw	GCCCATCTGTTTTCAATATTATTAAGAATG
P10-386	E5in1rv	GGTTCGGAACGATTTGGAAG
P10-389	E5in2fw	TAGAACAGTGTCTTGTGCAGATA
P10-390	E5in2rv	CTCCCCAACGGAACTG
P10-391	E5in3fw	TTAAAAAAGCTTTTGCTGACGTTGGTT
P10-392	E5in3rv	GAGCTGTCACAAATAGGCATC
P10-432	gWalkingAdaptorStrand1	GTAATACGACTCACTATAGGGCACGCGTGGTCGACGGCCCGGGCTGGT
P10-433	gWalkingAdaptorStrand2a	GATCACCAGCCCCT
P10-434	gWalkingAdaptorStrand2b	CCGGACCAGCCCCT
P10-435	gWalkingAdaptorStrand2c	AGCTACCAGCCCCT
P10-218	HRPA2_3UTR_rv	CCAGAGCTTGCCATTATGTG
P10-219	HRPA2_Cterm_fw	CACAATAGCGGTTGTTACCTC
P10-073	HRPA2_Cterm_rev1	CAACTTCCATTAACCTTCTTACAGTC
P10-116	HRPB1_Cterm_rev	CCAAATATTCTTAAGTAATGTTTCGAGAAC
P10-103	HRPB1_ex1_rev	GATCTGATCTTAGCTCGTTCAC
P10-106	HRPB1_ex2_fw	CTGTGATGCATCGATTTTGTTAG
P10-107	HRPB1_ex2_rev	CTGCGCATGATACGGTTC
P10-110	HRPB1_ex3_fw	CAGGAGGTCCTTCTTGG
P10-111	HRPB1_ex3_rev	GTCCAACATTACTAAAGCTGGC
P10-115	HRPB1_ex4_fw2	CAACTCGCTGCTCCATG
P10-114	HRPB1_ex4fw1	GGTCACACATTTGGTAAAAACCAATG
P10-104	HRPB1_in1_fw	GTGAACGAGCTAAGATCAGATC
P10-105	HRPB1_in1_rev	GCTGCATCTTTCTCTGTTCG
P10-108	HRPB1_in2_fw	GAACCGTATCATGCGCAG
P10-109	HRPB1_in2_rev	CAAGATCAAAAAATGCTTGTACGC
P10-112	HRPB1_in3_fw	GCCAGCTTTAGTAATGTTGGAC
P10-113	HRPB1_in3_rev	GTTACTAAAGTTGTATAGTCTGTCC
P10-075	HRPB1_Nterm_fw	CAACTTAAACCAATAAAAGATAAGTTTCCTC
P10-001	HRPC1A_ex1F	CGTTTTGCCTATAAAAGGATTC
P10-002	HRPC1A_ex1R	AAATGATAAATGTAATTAGACAGTATG
P10-003	HRPC1A_ex2F	GACAAAAATGTTACATTGTTGC
P10-004	HRPC1A_ex2R	GTGTTGATATGTAAAGTGACTATTTG
P10-005	HRPC1A_ex3F	CACATATTTTTCTCTTAACACATTG
P10-006	HRPC1A_ex3R	CTAAATACAACTATATCAAATGGTG
P10-008	HRPC1A_ex4R	GATAGAGATCTTCTCATGCTC

Number	Name	Sequence
P10-017	HRPC1B_ex1F	GGATTATATAAGATATGGACCTTAC
P10-018	HRPC1B_ex1R	CAAAACAAATTGACTCTTTGTATC
P10-019	HRPC1B_ex2F	GTTAGCTATGGATGTAATACATG
P10-020	HRPC1B_ex2R	GAACAGTTGTTGATACTAAATATAG
P10-021	HRPC1B_ex3F	CTTCCGAGTGACAAATTAATCTC
P10-022	HRPC1B_ex3R	CTAATTGAACTTATATCAAATGGTG
P10-023	HRPC1B_ex4F	CACCATTTGATATAAGTTCAATTAG
P10-024	HRPC1Bex4R	CTTCTCCTTCTCAAGTAACATC
P10-228	HRPC1C_ex1_rv	CACTTCCACGACTGCTTTG
P10-231	HRPC1C_ex2_fw	GGTTGTGACGCATCGATC
P10-232	HRPC1C_ex2_rev	CAGATTGTTGAGCTGCAATGG
P10-235	HRPC1C_ex3_fw	GCAGGAGGTCCTTCTTG
P10-236	HRPC1C_ex3_rv	GAGAGCAACGAGATCAGAAG
P10-239	HRPC1C_ex4_fw1	GGTCACACATTTGGTAAAAATCAATG
P10-240	HRPC1C_ex4_fw2	GAACTCAAGGAGAAATCAGGTTG
P10-188	HRPC1C_fw2	GCTTCATGCATCTTTTTCCAATG
P10-229	HRPC1C_in1_fw	CAAAGCAGTCGTGGAAGTG
P10-230	HRPC1C_in1_rv	GTTCGAAATGATGTTGTGTTGTC
P10-233	HRPC1C_in2_fw	CCATTGCAGCTCAACAATCTG
P10-186	HRPC1C_rv2	CACACTACACACCAATAAAGATATTC
P10-009	HRPC2_ex1F	CACTCAACTTCAAACCTAAC
P10-010	HRPC2_ex1R	GAAGTACTTAAACAGGTTTTTTACTAG
P10-011	HRPC2_ex2F	GATTTAACTATGAATATGGTAGTTG
P10-012	HRPC2_ex2R	CCTAAAAATTAAAATCAATAAGATGATATG
P10-013	HRPC2_ex3F	CATATCATCTTATTGATTTTAATTTTTAGG
P10-014	HRPC2_ex3R	GAGTTTATTAATGACTACAACTATAG
P10-016	HRPC2_ex4R	CTATAGTTGTAGTCATTAATAAACTC
P10-195	HRPE5_rv1	GATATATATTCCCAACATAATCACATAGAAC
P10-449	newC1CNtermSpecific1a	GTAACTCATTGATAATGATGTCCC
P10-450	newC1CNtermSpecific2	TCATTGATAATGATGTCCCGTACTATG
P10-451	newNNtermSpecific1a	GACAATTTGTAAAAGATTCGGGCAC
P10-453	newNNtermSpecific2	GTGCCCTAACCGCTGAAC
P08-483	pJET1.2fw	CGACTCACTATAGGGAGAGCGGC
P08-484	pJET1.2rv	AAGAACATCGATTTTCCATGGCAG
P07-514	RT-synPDI-rev	ACTTGGACGATAACTGGCTCTTTAG
P09-338	Zeocin:fw	GACTCGGTTTCTCCCGTGACT
P09-337	Zeocin_rv	CTGCGGAGATGAACAGGGTAA
P12022	23190F1	GGAACAACAAGAAGCAGAGAAGAGAGAG
P12023	23190F2	CGACTGAAACAACAAAAAATGGCAATG
P12024	23190F3	ATGGCAATGAGTTATTCGATACGTGTC
P12025	23190R1	GAGATAGTCTTAGGCATTCCACAAACC
P12026	23190R2	GTTATTTTAGATCATGGAAAGAGCTTCC
P12027	23190R3	GGATATGAAACTTGCGGTGTTTCTGG
P12028	04663F1	CAAAGCTCTATCATTATTTGCAACAAAC
P12029	04663F2	CTGATTAATGGCTGCAACAAGCTCTTC
P12030	04663F3	ATGGCTGCAACAAGCTCTTCTACTAC

Number	Name	Sequence
P12031	04663R1	GACTGAGCAAAAGCCTCAAAAAACAGG
P12032	04663R2	GTTTGGTTACTTGCAAAGGATTACAATC
P12033	04663R3	CTTGCAAAGGATTACAATCGCGATGG
P12034	06351F1	GATTACAAGATTTAAGATAGAAAATAATAAGATGG
P12035	06351F2	GATGGTTAGGGCAAATTTAGTGAGCG
P12036	06351F3	GCAAATTTAGTGAGCGTGATTCTGTTAATGC
P12037	06351R1	GGAGCATAAATACAAAAATCGGCCTAGGC
P12038	06351R2	GACATTATGACTCGAATTAATGACAGAGTAGG
P12039	06351R3	AAATCGGCCTAGGCTTAGTTAATAGTCC
P12040	05508F1	CTAAAAACACACTTGATCTTCTCTAAAACATCG
P12041	05508F2	CACTTGATCTTCTCTAAAACATCGAAACATAAATAC
P12042	05508F3	ATACAAGATGGGTTTGATTAGATCATTATGC
P12043	05508R1	ATCGGTTAATTAATTAATCGCAGAGCAAACC
P12044	05508R2	CACCACTGATTTTAATCGGTTAATTAATTAATCG
P12045	05508R3	CAAACCTTACGAATTTCCCCATTAGTCC
P12046	22489F1	CACTTGATCTTCTCTAAAACACTAAAATTATATATCC
P12047	22489F2	CACTAAAATTATATATCCAATATGGAGTTTGTTAG
P12048	22489F3	CCAATATGGAGTTTGTTAGATCATTATGC
P12049	22489R1	TCTGTTTATCACCACTGATTTTAATCGG
P12050	22489R2	GCAGAGCAAACCCTACGAATTTCC
P12051	22489R3	GATTTTAATCGGTTAATTAATTAACCGCAGAGC
P12195	23190seqF1	GAGAACAATCATCGATCCCGAAC
P12196	23190seqF2	GTCAACAAGCCTTTGTTGTCATCAATAACC
P12197	23190seqF3	CATCGGAATTGCGCATTGTCCGTC
P12198	23190seqF4	CAAGATCCAACCATGAACAAGTCTTTC
P12199	04663seqF1	CCATCGTACGCAGCACTATCCAGC
P12200	04663seqF2	GCAAGCTCTTCAATCCGACCCGAG
P12201	04663seqF3	TTGCCTCAGAGGCTTCCGTGTCTTTG
P12202	04663seqF4	CCTTCGAAGGCCTTAACAACATCAC
P12203	06351seqF1	GCTCTTCAAGCCGATCCCACTTTAGC
P12204	06351seqF2	CCACTTTAGCCGCAGGTCTTATACG
P12205	06351seqF3	CCTTTGGCAACCGTGGCTTCTCTCC
P12206	06351seqF4	GCAAGATGTTGTTGCTCTCTCTGG
P12207	05508seqF1	GCACCCGGAATATTGAGAATGC
P12208	05508seqF2	GCATTTCCACGACTGCTTCGTTCAAG
P12209	05508seqF3	CGTGACTCCGTCGCCGTTCAACAAC
P12210	05508seqF4	CAGTGCGCGTGGATCTCGATAC
P12211	22489seqF1	GGAATATTGAGAATGCATTTCCACG
P12212	22489seqF2	CGACTGCTTCGTTCTAGGTTGTGACG
P12213	22489seqF3	CGTGACTCCGTCGCCGTTCAACAAC
P12214	22489seqF4	CGACACCGGAAGTGGAACCAC
P12320	05508seqF5	ATGTGAGAAACGTTTTACGTGCGTG
P12321	05508seqR5	TTAACATGAAGAGTTTCTCAACG
P12322	05508seqR6	CATGAAGAGTTTCTCAACGTTAATTTTTCG
P11114	03523noSSfw1	ATATCTCGAGAAGAGAGAGGCCGAAGCTAGACTGACTACCAACTTCTAC
P11115	03523noSSrv1	TATAGCGGCCGCATTAGTTGATTG

Number	Name	Sequence
P11116	04663noSSfw1	ATATCTCGAGAAGAGAGAGGCCGAAGCTCAGTTGAACGCAACCTTCTAC
P11117	04663noSSrv1	TATAGCGGCCGCATTACTTACACAAG
P11118	05508noSSfw1	ATATCTCGAGAAGAGAGAGGCCGAAGCTCAAGCTATCTCCATTTCCATTAC
P11119	05508noSSfw2	ATATCTCGAGAAGAGAGAGGCCGAAGCTACCATCAGAATTGGTTTCTACCTTAC
P11120	05508noSSrv1	TATAGCGGCCGCATTAGTTGATAGC
P11121	06351noSSfw1	ATATCTCGAGAAGAGAGAGGCCGAAGCTTTCCCATTCCACGCCAGAGGTTTG
P11122	06351noSSrv1	TATAGCGGCCGCATTAGTTGATGGTTC
P11123	23190noSSfw1	ATATCTCGAGAAGAGAGAGGCCGAAGCTAAGAAGCCACGTAGAGACGTTC
P11124	23190noSSfw2	ATATCTCGAGAAGAGAGAGGCCGAAGCTGGTTTGTCATGGAACTTCTAC
P11125	23190noSSrv1	TATAGCGGCCGCATTAGATCATAGAAAG
P11126	22489noSSfw1	ATATCTCGAGAAGAGAGAGGCCGAAGCTCAGGCTGCCGCTAGAAGACCAG
P11127	22489noSSfw2	ATATCTCGAGAAGAGAGAGGCCGAAGCTGGTACTAGAATTGGTTTCTACTTAAC
P11128	22489noSSrv1	TATAGCGGCCGCATTAGTTGACTGCTG
P11129	04791noSSfw1	ATATCTCGAGAAGAGAGAGGCCGAAGCTAGATTGACTACCAACTTCTACTCTAAG
P11130	04791noSSrv1	TATAGCGGCCGCATTAATTGATAG
P11131	06117noSSfw1	ATATCTCGAGAAGAGAGAGGCCGAAGCTGACGACGAGTCCAACTACGGTG
P11132	06117noSSfw2	ATATCTCGAGAAGAGAGAGGCCGAAGCTAAGTTGTTCCCTGGATTCTAC
P11133	06117noSSrv1	TATAGCGGCCGCATTAAGAGTTGATC
P11134	17517noSSfw1	ATATCTCGAGAAGAGAGAGGCCGAAGCTAGAAGACCTAGAGTTGGTTTC
P11135	17517noSSfw2	ATATCTCGAGAAGAGAGAGGCCGAAGCTAGACCTAGAGTTGGTTTCTAC
P11136	17517noSSrv1	TATAGCGGCCGCATTAGTTGATGGC
P11137	08562noSSfw1	ATATCTCGAGAAGAGAGAGGCCGAAGCTGACAAATCCTACGGTGGAAAG
P11138	08562noSSfw2	ATATCTCGAGAAGAGAGAGGCCGAAGCTAAGTTGTTCCCAGGTTTCTAC
P11139	08562noSSrv1	TATAGCGGCCGCATTAAGAGTTGATC
P12518	04663R6	ACACAAATGATTCAGCTTAGGAG
P12519	04663R7	GATTCAGCTTAGGAGTAATTATTCATGTATC
P12520	04663R8	CATGTATCATAAACAAACGGTG
P12521	04663R9	GTATCATAAACAAACGGTGAAACTGATCC
P12522	C3F1	GATTCACACCATCAGCCACAC
P12523	C3F2	CGCCTTAGCTAGATTCACACC
P12524	C3F3	GTTCCTTCTACGCCTTAGC
P12525	C3F4	CTCCAGCTACTCTATATAGTGTTCC
P12526	C3F5	CACATATAGAATAGAGGCCAAAAGG
P12527	C3F6	CATCGCCTCTCAAATATCAGTGC
P12528	C3F7	CTTCGATTTGGCTAATACAGCTCTTC
P12529	C3F8	CAACTGACAAACCATAAAAACTTAAACATGC
P12530	C3F9	GAATTAGGGGTATGGAGAACGATG
P12531	C3R1	CGACATGCATGTTCCCACACATTTTATG
P12532	C3R2	GTGAGAGCTTTTATTTAGTCGACATGC
P12533	C3R3	CACAAGTCATAACTCGTGAGAGC
P12534	C3R4	CAGTTGTAATCTCACAAGTCATAACTCG
P12535	C3R5	CTTTTCTTTCCTTTGGTTTTTTCAGTTG
P12536	C3R6	GAGTCGAGAAGAGCTCTTGG
P12537	C3R7	AAAGTCATGATTTTTTCGTTTTACTAATTCATG
P12538	C3R8	GCTTGAAGACATTTTGTTTGAGATGG
